# Looking Back While Reading Ahead: Regressive Fixations and Temporal Anticipation in Music Sight-Reading

**DOI:** 10.3390/jemr19050102

**Published:** 2026-09-15

**Authors:** Michel A. Cara, Divna Mitrovic

**Affiliations:** Department of Pedagogy, Music Institute, Faculty of Philosophy and Education, Pontificia Universidad Católica de Valparaíso, Viña del Mar 2520000, Chile

**Keywords:** regressive fixations, music sight-reading, eye movements, eye–hand span, temporal anticipation, rhythmic accuracy, visuomotor integration, predictive processing, blink count, embodied cognition

## Abstract

This study examined how regressive fixations relate to temporal anticipation and unfolding temporal dynamics during music sight-reading. Eye-movement, performance, and respiratory data were recorded from 28 flutists performing three scores differing in musical and metrical structure; performance recordings were MIDI-aligned with eye-movement and respiratory measures. Regressive fixations were defined as fixations following regressions toward previously viewed score notes. A participant-grouped random forest model explained 60% of the out-of-fold variance in regressive-fixation count, highlighting fixation count, mean fixation duration, and time-based eye–hand span as leading predictors. Mixed-effects analyses showed that time-based eye–hand span was the most consistent predictor, while performance duration and mean fixation duration provided additional information. Rhythmic accuracy moderated the relationship between temporal anticipation and regressive-fixation count, with a stronger positive association in more accurate performances (β = 1.062, 95% CI [0.282, 1.842], *p* = 0.008). This interaction remained significant when regressive-fixation count was modeled relative to performance duration and after controlling for mean fixation duration. An additional analysis found no evidence that shorter fixation durations attenuated the association between more regressions and longer relative performance duration. These findings suggest that regressive fixations are associated with temporal anticipation during music sight-reading and may inform cross-domain research on the role of regressive eye movements in reading comprehension.

## 1. Introduction

Music sight-reading involves performing written music without prior practice. From an embodied-cognition perspective, sight-reading constitutes a dynamic perception–action system in which visual information is continuously linked to predicted auditory and motor outcomes [1,2]. Performers must transform a continuously unfolding visual code into precisely timed actions while distributing attention between current information and upcoming musical events. Anticipatory mechanisms, particularly the eye–hand span (EHS), are therefore central to fluent sight-reading and musical performance [3,4]. Sight-reading thus provides a valuable framework for examining how eye movements alternate between looking ahead to prepare forthcoming actions and returning to previously viewed notation through regressions when additional processing is required [5,6]. Although regressions have been extensively investigated in text reading, they have been examined less systematically in music reading, and their contribution to sight-reading performance remains unclear. Current reviews of music-reading research suggest that regressions may reflect local re-inspection and ongoing processing demands [7,8]. However, their relationship with EHS has not been clearly established, and no previous study has examined how regressions relate simultaneously to EHS and rhythmic accuracy during sight-reading, leaving unresolved the extent to which they are involved in temporal processing during music reading.

### 1.1. Reading Ahead and Regulating the Perception–Action Interval

Anticipation in music reading has most often been quantified through the EHS, defined as the distance between the currently fixated location in the score and the musical event being performed. The eyes typically precede musical execution, allowing upcoming material to be encoded before it must be performed [3,9,10]. EHS can be expressed spatially, in notes or beats, or temporally, as the interval between fixation and execution [4,11,12]. These forms are related but not interchangeable. A note-based EHS (EHSN) estimates the number of notes by which gaze precedes performance, whereas a time-based EHS (EHST), also referred to as the time-index, represents the interval for which visually acquired information must remain available before motor realization.

EHS should not be viewed as a fixed capacity or a linear indicator of proficiency, but as an elastic visuomotor measure that varies with task demands. Although skilled musicians generally exhibit larger spans, EHS varies with tempo, local note density, score structure, and motor requirements [13,14,15]. Anticipation expands when upcoming material can be prepared in advance but contracts when processing demands arise near the point of execution [16], while expert musicians adapt EHS, fixation duration, and saccadic amplitude more flexibly to local score complexity [17]. Accordingly, EHS is better understood as an adaptive index of visuomotor coordination than as a fixed marker of reading ability [14,18].

Empirical evidence indicates that EHS is closely related to temporal characteristics of performance. EHS varies systematically with playing tempo and musical complexity [13], while controlled-tempo studies show that anticipatory gaze and saccadic behavior adapt to temporal constraints and local notational demands [16]. Likewise, longer performance duration has been associated with more fixations, shorter EHS and reduced performance fluency in advanced pianists [19].

The effectiveness of anticipation also depends on how notation is transformed into coordinated action. Expertise facilitates chunking and the use of structural regularities, allowing several notated elements to be represented as functional units [19,20,21,22], whereas local incongruities and visually demanding features modify gaze behavior and predictive processing [23,24,25]. Consequently, studies using stimuli with limited syntactic coherence may underestimate the role of these structural processes [26].

A functional comparison can be drawn with oral reading, where the eye–voice span (EVS) quantifies the temporal and spatial relationship between eye movements and vocal production. Inhoff et al. [27] showed that readers regulate EVS by adjusting viewing time and, when necessary, by returning to previously read text. Laubrock and Kliegl [28] reported that larger spatial EVS is associated with increased probabilities of refixations and regressions, whereas Easson et al. [29] found a positive association between temporal EVS and regressive-fixation count in serial naming tasks.

### 1.2. Rhythmic Accuracy as Temporal Performance

During sight-reading, musicians must preserve notated duration and onset relations while continuing to decode forthcoming material. Rhythmic accuracy can therefore be considered a performance-based indicator of temporal-musical integration. Because performances may differ in overall tempo while preserving notated duration ratios, or maintain a similar tempo while locally altering rhythmic relations, duration-based score–performance comparisons provide an appropriate measure of departures from the notated temporal structure [30,31]. Rhythm production is guided by hierarchical metrical expectations [32,33,34]. Regular temporal structures generally facilitate preparation, whereas syncopation, unequal subdivisions, displaced accents, and other departures from expected patterns require continuous updating of temporal predictions [35,36]. However, rhythmic difficulty depends not only on score structure but also on prior experience, as non-isochronous meters can support precise coordination when their grouping principles are culturally familiar [37].

Consistent with this framework, sight-reading accuracy has been related to tempo, score complexity, motor constraints, working memory, and musical expertise, while tempo additionally constrains visual processing time and the coordination between gaze and execution [13,16,30,38,39]. Much of this literature has focused on global performance measures or pitch-related errors. To our knowledge, no previous study has examined rhythmic accuracy in relation to regressions during sight-reading.

### 1.3. Regressive Eye Movements and Integrative Mechanisms in Music Reading

A regression is a backward-directed saccade toward a previously viewed location in the score, and the fixation following this movement is referred to as a regressive fixation. In text reading, regressions are considered functionally heterogeneous, serving oculomotor correction, reinspection of insufficiently encoded material, ambiguity resolution, or higher-level integration across sentences and discourse [40,41]. The Information Gathering Framework further proposes that regressions occur when the information accumulated during the current fixation is insufficient to support continued forward reading, either due to insufficient encoding or failure of integration into the developing representation [42]. Although linguistic and musical reading are not directly equivalent, this distinction provides a useful framework for interpreting regressions as reflecting either unresolved processing demands or functional information reacquisition.

Evidence from music reading is broadly consistent with both interpretations, although the functional significance of regressions is no yet fully understood. Early studies described regressions as returns to previously inspected notation to obtain additional information [43,44,45], whereas Perra et al. [8] proposed that regressions provide renewed access to previously inspected notation during ongoing performance, emphasizing their role in information updating.

The determinants of regressions in music reading are still emerging within the music cognition literature, although further empirical work is required to clarify their functional specification. During silent score reading, less experienced musicians produced more regressions than experts and were more affected by the absence of phrase markings [5]. In a silent reading task, Cara and Gomez Vera [46] showed that regressions within and across musical phrases varied with musical style and between first-pass and re-reading stages, suggesting both local reinspection and broader integrative functions. More recently, Drai-Zerbib et al. [47] reported that patterns of regressive fixations can also serve as predictors of musicians’ expertise level, distinguishing intermediate from advanced performers. Regressions also appear sensitive to task and notational demands. Wurtz et al. [12] observed a tendency toward more regressive fixations in the structurally more complex violin sonata by Telemann accompanied by longer fixation durations and reduced note-based anticipation. In erhu sight-reading, notation type and score difficulty influenced regressive-saccade size, while increasing difficulty was associated with greater fixation duration and reduced EHS [6]. More generally, fixation and saccadic behavior adapt to local score complexity, suggesting that the functional significance of regressions depends on the temporal, structural, and notational contexts in which they occur.

### 1.4. Oculomotor and Physiological Regulation During Music Performance

Mean fixation duration reflects how visual processing time is distributed and varies with learning and task demands. Fixations become shorter as music reading strategies develop [18,48], while note density and repeated exposure also influence fixation duration [49]. Increasing difficulty has been associated with shorter, more frequent fixations in woodwind sight-reading [50], a pattern also observed across structurally demanding musical passages [15,51]. Perra et al. [17] further showed that local score complexity increases fixation duration more strongly in less-expert than in more-expert readers.

Blink activity provides complementary information about fluctuations in visual engagement and attentional state [52,53]. In music reading, blink patterns have been associated with musical expertise [47]. Exploratory evidence further suggests that blinks tend to align with musical phrase boundaries, indicating that they may adapt to music structure during performance [54]. More recent work in flute practice has related blink activity to learning strategies, fixation duration, anticipatory behavior, and physiological regulation [18].

Pupil diameter offers a further measure of processing demands and arousal. Pupil responses vary with harmonic incongruity [24] and structural complexity [25]. They are also sensitive to locally demanding notational features [55] and to affective or bodily engagement in musical tasks [56]. A longitudinal case study that followed an expert cellist from initial sight-reading to later performance further showed that peaks in pupil dilation coincided with score locations identified by the performer as technically demanding [57].

Respiratory regulation is integral to flute performance, supporting sound production, phrasing, and temporal coordination through task-dependent inspiratory and expiratory control [58,59]. Respiratory timing varies with phrase structure and performance context [60], and musicians tend to breathe at similar locations across repeated performances [61]. Respiratory patterns have also been associated with affective state and music performance anxiety [62]. More directly relevant to visual anticipation, respiratory coordination has been associated with both temporal and note-based EHS during flute practice [63]. Although previous studies have not directly examined blinking, pupil dynamics, or breathing in relation to regressions, these measures may help characterize the broader oculomotor and physiological context in which regressions occur.

### 1.5. The Present Study

Musical phrases provide a meaningful scale for examining regressions because they organize melodic and rhythmic information and define structural boundaries across which regressions may serve different functional roles [44,46,64]. The present study investigates regressive fixations during flute sight-reading. It examines associations with temporal and note-based anticipation, indexed by EHST and EHSN, respectively, as well as with performance duration and rhythmic accuracy. It further tests whether rhythmic accuracy moderates the relationship between EHST and regressive fixations and whether this relationship persists after accounting for mean fixation duration. An exploratory participant-grouped random forest evaluates whether a broader set of oculomotor, respiratory, and performance-related variables predicts regressive-fixation count. By combining these approaches, the study examines how regressive fixations are related to anticipatory processes across temporal and note-based dimensions, and how rhythmic accuracy modulates these relationships during music sight-reading. Preliminary unpublished findings from a related study conducted by our research group further suggested that temporal and note-based components of anticipation may respond differently to temporal and metrical demands.

### 1.6. Hypotheses

Given the limited evidence on regressive fixations in music sight-reading, the study combined exploratory multivariate prediction with hypothesis-driven mixed-effects analyses.

H1 predicted that regressive-fixation count would be predicted above a mean-only baseline from anticipatory, performance, oculomotor, physiological, and score-related variables using participant-grouped cross-validation. Anticipatory measures were expected to contribute to the prediction of regressive-fixation count, but no a priori ranking of individual predictors was specified.

H2 predicted that regressive-fixation count would be associated with both temporal and note-based components of anticipatory processing. Given the limited previous evidence, no directional prediction was made regarding their relative contributions. Accordingly, the associations of both components were hypothesis-driven, whereas their relative contributions across the progressively adjusted models were interpreted exploratorily.

H3 predicted that rhythmic accuracy would moderate the relationship between temporal anticipation and regressive-fixation count. Because previous evidence did not justify a precise directional prediction, the hypothesis concerned the presence rather than the direction of moderation.

Performance duration was included as a covariate to account for differences in the duration of the analyzed musical phrases. Mean fixation duration was additionally included in an exploratory sensitivity analysis as a general oculomotor measure that may be associated with both task demands and regressive fixations. This analysis examined whether the associations of regressive fixations with temporal anticipation and rhythmic accuracy remained after controlling for mean fixation duration.

## 2. Methods

### 2.1. Participants

Twenty-eight flutists (13 women), aged 18–46 years (*M* = 27.28, *SD* = 6.82), were included in the combined dataset. Participants were advanced students or active professional musicians recruited in Valparaíso and Santiago, Chile, and reported an average of 13.75 years of flute experience (*SD* = 2.29). All participants gave written informed consent and received compensation. After preprocessing and exclusions, the final analytical dataset comprised 409 phrase-level observations.

### 2.2. Musical Materials

The analysis included three single-line flute scores selected to represent contrasting rhythmic, structural, and technical demands. The Koechlin excerpt (KOE) comprised 22 measures from the second movement of the *Sonata for Flute and Piano*, Op. 52, organized into four phrases (measures 1–7, 7–12, 13–18, and 18–22). It uses a predominantly regular ternary organization; French verbal indications were removed from the displayed score. The Briccialdi excerpt (BRIC) comprised the first 25 measures of the first-flute part from the second movement of *Duo Concertante for Two Flutes in F Major*, No. 2, Op. 100. It contained a one-measure chromatic opening followed by six lyrical phrases, yielding seven analytical phrases in total: measure 1; measures 2–5, 6–9, 10–13, 14–17, 18–21, and 22–25. Anacrusic notes were assigned to the phrase they introduced, including cases of phrase elision. The third score (DEV) consisted of an adaptation of Jonče Hristovski’s *Makedonsko devojče*, a Macedonian melody in 7/8 m. It comprised 32 measures divided into four eight-measure phrases. The average number of notes per musical phrase was 26.50 for DEV (*SD* = 0.58; range = 26–27), 31.25 for KOE (*SD* = 5.91; range = 23–37), and 28.86 for BRIC (*SD* = 6.89; range = 22–42). Tied notes were counted separately for this calculation. The unequal metrical grouping in DEV contrasts with the more familiar regular organization of KOE and the predominantly lyrical organization of BRIC. Participants’ prior familiarity with the musical materials was assessed before recording. None reported having previously performed KOE or DEV, whereas one participant reported limited prior exposure to BRIC.

**Figure 1:**
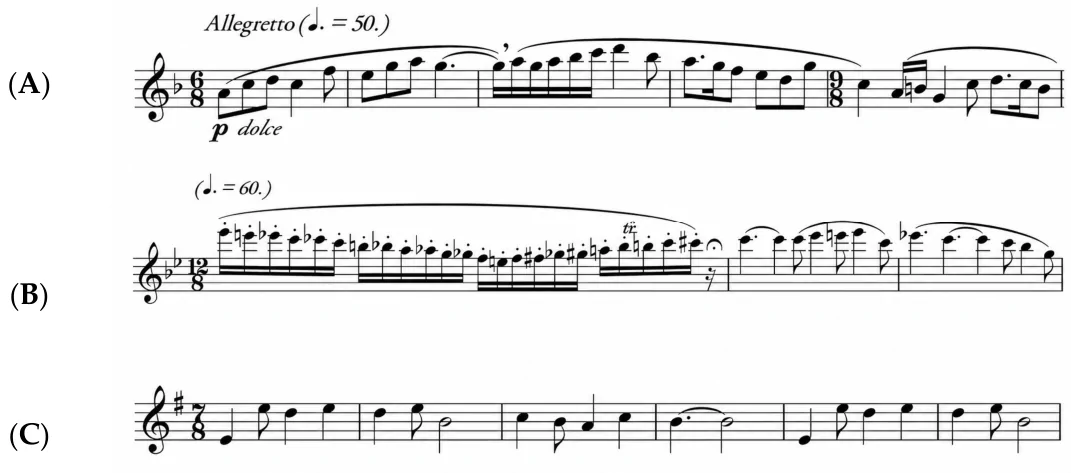
Opening measures of the three musical scores used in the sight-reading task: (**A**) Koechlin’s *Sonata for Flute and Piano*, Op. 52 (KOE); (**B**) Briccialdi’s *Duo Concertante for Two Flutes in F Major*, No. 2, Op. 100 (BRIC); and (**C**) Hristovski’s *Makedonsko devojče* (DEV).

All scores were displayed as JPEG images (1148 × 1080 pixels; 300 pixels/inch) on a 1920 × 1080 monitor. Representative excerpts from the different scores are shown in Figure 1.

### 2.3. Apparatus and Signal Synchronization

Eye movements were recorded binocularly with a Tobii TX300 remote eye tracker at 250 Hz (4 ms temporal resolution) and processed in Tobii Pro Lab 1.2. Participants sat approximately 600 mm from the display. A standard nine-point calibration was completed before each task; calibration was repeated when inspection indicated insufficient spatial accuracy. Across sessions for which calibration summaries were available, median calibration accuracy and precision were 0.550° (IQR [0.455°, 0.980°]) and 0.090° (IQR [0.075°, 0.110°]) for BRIC, 0.580° (IQR [0.415°, 0.770°]) and 0.090° (IQR [0.070°, 0.130°]) for KOE, and 0.595° (IQR [0.475°, 0.935°]) and 0.110° (IQR [0.070°, 0.133°]) for DEV, respectively. For comparison with the spatial separation between adjacent notes, center-to-center Euclidean distances between consecutive noteheads within each staff system were calculated from the note coordinates used for fixation assignment. These distances were converted to degrees of visual angle under the experimental viewing conditions. Median consecutive-note spacing was 0.622° (IQR [0.516°, 0.809°]) for BRIC, 0.777° (IQR [0.653°, 1.040°]) for KOE, and 0.993° (IQR [0.829°, 1.226°]) for DEV.

Flute performances were captured with a Sennheiser e914 condenser microphone (Sennheiser, Wedemark, Germany) connected to a PreSonus StudioLive 16.0.2 digital mixer (PreSonus Audio Electronics, Inc.: Baton Rouge, LA, USA) at 44.1 kHz. Thoracic and abdominal movements were recorded with two SA9311M respiration sensors connected to a ProComp 5 Infiniti unit (Thought Technology, Montreal, QC, Canada; 256 Hz). The bands were positioned around the thorax and abdomen using consistent anatomical landmarks. Ambient illumination was maintained at 120 lux and verified at the participant’s head position with a Mastech MS6612D lux meter (Mastech, Dongwan, China).

A dedicated C++ application acquired the ProComp signals through the manufacturer’s API. TTL pulses transmitted by the eye tracker and the ProComp decoder were recorded on separate mixer channels together with the flute signal. Custom MATLAB routines (R2023b; The MathWorks, Inc., Natick, MA, USA) aligned eye-tracking, respiration, and audio data to a common timeline. The small linear timing drift previously observed in the ProComp recordings was corrected by regression before alignment.

### 2.4. Design and Procedure

The study employed a within-participant sight-reading design in which each musician performed the three musical scores under sight-reading conditions. Recordings took place in the Language and Cognition Laboratory at the Pontificia Universidad Católica de Valparaíso. Participants first completed a questionnaire concerning musical training, sight-reading experience, and prior familiarity with the stimuli. They then performed the scores while seated, which reduced movement artifacts and standardized posture because respiratory mechanics may vary with body position [65].

The present study is based on data collected as part of a broader experimental protocol. The three scores were presented according to a fixed/counterbalanced order: DEV was always presented first, whereas the order of BRIC and KOE was counterbalanced across participants. Although the complete protocol included repeated performances of KOE and additional practice and performance conditions for BRIC, only the initial sight-reading performance of each score was analyzed in the present study. This ensured that all three scores were compared under equivalent sight-reading conditions. Before the initial performance, participants were allowed up to approximately 20 s to silently inspect the displayed score. All procedures were conducted in accordance with the ethical standards of the institutional Ethics Committee.

### 2.5. Data Preprocessing and Variable Construction

Eye-tracking data were available for 28 participants for DEV and for 27 participants for both BRIC and KOE, as two participants did not complete all experimental conditions.

#### 2.5.1. Performance Data

Flute recordings were converted from WAV to MIDI using a custom MATLAB application. After DC-offset correction, the audio was divided into 10 ms frames for intensity and pitch estimation by short-time autocorrelation. MIDI data were processed with the MIDI Toolbox [66] and written using KaraokeMidiJava.jar [67]. Conversion errors were identified with a score–performance alignment procedure adapted from Dynamic Matching Performance to Notation [68], and the remaining mismatches were corrected manually in a MATLAB MIDI editor. The aligned files also identified additions, substitutions, and omissions.

Performance duration (PerfD) was defined as the elapsed time required to execute each musical phrase. Tempo was expressed in beats per minute using the eighth note as the reference unit and was calculated from the score-based distance between consecutive onsets and the corresponding performed inter-onset interval.

Rhythmic accuracy was derived from the ratio between each performed note duration and its score-based MIDI duration; omitted and added notes were not included. Because raw ratios treat equivalent proportional deviations above and below 1 asymmetrically, the ratio was transformed as exp(−|log(r)|), where r is the performed-to-reference duration ratio. The resulting index ranges from 0 to 1, with values approaching 1 indicating greater durational accuracy. Phrase-level rhythmic accuracy was obtained by averaging valid note-level values. The original ratio was retained alongside the transformed index for subsequent analyses.

#### 2.5.2. Fixations and Note Assignment

Raw gaze samples were classified with the Tobii I-VT fixation filter using a velocity threshold of 30°/s, a minimum fixation duration of 60 ms, and no automatic gap interpolation. Fixations longer than 2000 ms were excluded as extreme observations [69]. Consecutive records assigned to the same score position were collapsed into a single fixation event. Only fixations within the boundaries of the displayed stimulus were retained. Each retained fixation was assigned to the nearest notehead based on Euclidean distance between the fixation and notehead coordinates. No maximum fixation-to-note distance was imposed in the main analysis. Thus, fixations between staves or in non-notated regions, including those approximately equidistant from neighboring noteheads, were assigned to the nearest notehead. The note being performed at the time of each fixation was determined from the aligned MIDI onset and offset intervals; when a fixation fell between intervals, the temporally nearest valid note event was used.

To assess robustness to spatial assignment uncertainty, a sensitivity analysis was conducted using maximum fixation-to-note distances of 1.0° and 0.6°. The 0.6° threshold provided a comparatively stringent criterion, approximately corresponding to the magnitude of the median calibration accuracy across the three scores, whereas 1.0° provided a more permissive spatial criterion. For each threshold, EHST and EHSN were recalculated after applying the corresponding spatial-assignment criterion, and regressive-fixation counts were restricted using the same criterion.

#### 2.5.3. Pupil Preprocessing

Pupil data were preprocessed separately for each eye. Samples were treated as missing when the eye was not detected (Tobii validity code 4), pupil diameter was unavailable or exceeded ±3 *SD* from the trial mean, or abrupt sample-to-sample changes exceeded ±3 *SD*. To reduce eyelid-related artifacts, missing segments were extended by 20 ms before onset and after offset [70]. Missing values were reconstructed from the fellow eye when available; otherwise, boundary gaps were replaced using the nearest valid 1 s window, and internal gaps were linearly interpolated. The resulting signal was smoothed with a third-order low-pass Butterworth filter (2 Hz), following current recommendations to treat artifact rejection, interpolation, and smoothing as separate preprocessing steps [71]. Mean pupil diameter was then calculated for each musical phrase and retained as a candidate oculomotor predictor.

#### 2.5.4. Blink Detection

Potential blinks were initially identified from binocular periods of pupil-data loss. Within each eye, adjacent loss segments separated by less than 80 ms were merged, and only temporally overlapping segments in both eyes were classified as blinks. Events shorter than 50 ms or shorter than 20% of the participant’s mean blink duration were removed. Long events exceeding the participant mean by more than three standard deviations were also excluded; when within-participant variability was insufficient for this criterion, 600 ms was used as the upper limit. Blink count and mean blink duration were calculated per phrase. This conservative binocular procedure reduces the risk of treating brief tracking failures as physiological blinks, an issue emphasized in recent methodological work [72].

#### 2.5.5. Respiratory Preprocessing

Thoracic and abdominal signals were inspected by continuous wavelet analysis to identify the frequency content of movement and recording noise. They were then filtered with a third-order IIR Butterworth band-pass filter using cutoffs of 0.08–10 Hz for the thoracic channel and 0.1–3 Hz for the abdominal channel, consistent with Sánchez-Solís et al. [73]. Peaks and troughs exceeding two standard deviations were removed before min–max normalization.

Respiratory cycles were detected with the SpeechBreathingToolbox [74], with participant-specific parameter adjustments. Thoracic and abdominal traces were synchronized, and low-amplitude thoracic fluctuations without a corresponding respiratory pattern were not considered inhalation onsets. When a valid thoracic inhalation lacked a clear abdominal counterpart, the abdominal signal was reconstructed using the minimum-to-maximum interval observed within the same thoracic inhalation. Phrase-level respiratory measures included breathing rate, thoracic and abdominal movement amplitudes, and the thoracic-to-abdominal performance ratio (Th–A ratio), calculated as the maximum thoracic amplitude divided by the maximum abdominal amplitude.

#### 2.5.6. Anticipation Measures

Eye–hand span was calculated from the synchronized fixation and MIDI sequences using complementary note- and time-based definitions [16]. Note-based EHS (EHSN) followed the forward-projective approach: for each performed note, it was the largest positive distance, in score notes, between the note being played and the farthest upcoming note fixated during its execution. Time-based EHS (EHST) followed the single-item-lag approach: it was the interval in seconds between the first anticipatory fixation on a target note and the subsequent onset of that same note. Only positive anticipatory events fewer than 20 notes ahead of execution were retained. Phrase-level values were calculated from valid events.

Subsequent regressive fixations did not reset or modify an EHSN or EHST value once determined for a given note. Phrase-level values were calculated by averaging the EHSN and EHST values obtained for individual notes within each participant and phrase.

#### 2.5.7. Regressive Fixation Measures

Regression measures were computed from successive valid fixations after both had been assigned to score notes. A standard regression was defined as a regressive saccade from one fixation location to an earlier fixation location within the score. Accordingly, regressions were identified from the direction of the saccade between successive fixations rather than from the location of gaze relative to the note being performed. This definition corresponds to the conventional characterization of regressions in music-reading research (see Introduction).

For each musical phrase, regressive-fixation count (RegC) was computed as the total number of regressive fixations within the phrase and served as the primary outcome measure in all predictive analyses. Three additional regression-related measures were calculated to provide complementary descriptive information but were not used as outcomes in the predictive models: (1) mean regressive-fixation duration, calculated as the average duration of all regressive fixations within the phrase; (2) mean regression size, defined as the mean distance, in notes, between the score locations assigned to the fixation immediately preceding a regression and the regressive fixation; and (3) phrase-level regression proportion, calculated as the proportion of regressive saccades relative to all valid saccades linking consecutive fixations. Thus, each regression was assigned to the phrase containing the regressive fixation rather than to the phrase containing the fixation immediately preceding the regression. The fixation immediately preceding the regression was used only to determine the direction and size of the corresponding regressive saccade, whereas the regression itself was attributed to the phrase containing the regressive fixation, even when the preceding fixation belonged to a different musical phrase.

#### 2.5.8. Experience and Emotional State Variables

Flute experience was expressed as years of playing or formal training, according to the available questionnaire record. Positive and negative emotional states were derived from six items of the PERMA Profiler [75], using the Chilean adaptation by Cobo-Rendón et al. [76]. Three items were averaged for each emotional state dimension. These participant-level variables were available as candidate predictors but were not used as substitutes for participant identity.

### 2.6. Statistical Analysis

#### 2.6.1. Random Forest Regression

Random forest regression [77] was used to assess the out-of-sample predictability of phrase-level regressive-fixation count and to identify variables carrying multivariate predictive information. Candidate predictors represented anticipation (EHST and EHSN), performance characteristics (performance duration, rhythmic accuracy, and errors), general oculomotor behavior (fixation count and duration, pupil dynamics, and blink count and duration), respiratory activity (Th–A ratio, thoracic and abdominal movement amplitudes, and breathing rate), musical experience, positive and negative emotions, and score type. Participant identity was not included as a predictor. Tempo was excluded to limit redundancy with performance duration, which matched the phrase-level unit of analysis and represented the total execution time available for regressive fixations to occur. Each random forest comprised 600 regression trees, with a minimum leaf size of five observations. Predictive performance was evaluated using five-fold cross-validation grouped by participant, ensuring that all phrase-level observations from the same participant were assigned to the same fold. The cross-validation procedure was repeated 5 times using different participant-level fold allocations. Results were averaged across the participant-level validation runs. Out-of-fold performance was quantified using R^2^, RMSE, and MAE. Predictor importance was estimated using permutation importance and summarized across repeated model fits.

Shared predictive information among related predictors was examined using grouped permutation. Predictors were permuted individually and in two conceptually defined blocks: performance duration, rhythmic accuracy, and EHST; and fixation count and mean fixation duration. Thirty permutations were performed per predictor or block, and changes in out-of-fold R^2^, RMSE, and MAE quantified individual and joint predictive contributions. Because fixation count may partly index opportunities for observing regressions, the random forest was also refitted without fixation count.

#### 2.6.2. Mixed-Effects Models

The mixed-effects modeling strategy comprised a primary count-model sequence, a fixation-duration sensitivity model, supplementary robustness models, and one exploratory temporal-compensation model.

Score was included a priori as an effects-coded categorical control factor (sum-to-zero contrasts), such that score coefficients represented deviations from the model-based average across score levels rather than comparisons with a single reference score. Random intercepts for participant and score–phrase accounted for repeated observations from the same musician and for variation shared across performances of the same musical phrase.

Model distributions and link functions were selected according to the outcome: Poisson log-link models for regressive-fixation counts and regression rates, binomial logit-link models for regression probabilities, and a Gaussian identity-link model for the exploratory temporal-compensation analysis.

EHST, EHSN, performance duration, rhythmic accuracy, and mean fixation duration were grand-mean centered but not standardized. Multicollinearity was assessed using variance inflation factors.

Missing data were handled using participant-phrase complete-case analysis, with no imputation. The primary count models, the fixation-duration sensitivity model (SensFixD), the rate-based robustness models, and the regression-probability robustness models were fitted to the same 409 complete participant–phrase observations; the exploratory temporal-compensation model used complete cases for its model-specific variables.

##### Primary Count-Model Sequence

Four sequential Poisson generalized linear mixed-effects models examined regressive-fixation count. The sequence first tested temporal and note-based anticipation, then added performance duration and rhythmic accuracy, and finally tested whether rhythmic accuracy moderated the association between EHST and regressive-fixation count. Model 4 was the focal inferential model because it represented the complete theoretically specified interaction model. To characterize the EHST × rhythmic-accuracy interaction in the primary Model 4, conditional simple slopes of EHST were estimated at the 25th percentile, mean, and 75th percentile of the observed rhythmic-accuracy distribution. Confidence intervals for the simple slopes were computed from the fixed-effect covariance matrix of the fitted model. To assess sensitivity for detecting the focal EHST × rhythmic-accuracy interaction under the observed multilevel design, 500 datasets were simulated from the fitted Model 4 while preserving the observed predictor values and participant- and score–phrase-level grouping structure. Model 4 was refitted to each simulated dataset, and sensitivity was estimated as the proportion of simulations in which the interaction was detected at α = 0.05; the proportion of estimates retaining the observed positive direction was also examined. A fixation-duration sensitivity model, SensFixD, reproduced Model 4 while additionally controlling for mean fixation duration. Additional sensitivity models reproduced Model 4 under the two spatial-assignment thresholds described above (0.6° and 1.0°). A further regression-size sensitivity model reproduced Model 4 after excluding one-note regressions (see Supplementary Table S1b). Model adequacy was evaluated by examining overdispersion using the Pearson dispersion ratio and potential excess zero inflation by comparing the observed number of zero-count observations with the number expected under the fitted Poisson model. Full specifications and analytical purposes are summarized in Table 1.

##### Supplementary Robustness and Exploratory Analyses

The random forest analysis was not used for automatic variable selection. Supplementary robustness analyses tested whether the principal findings remained after accounting for performance duration as temporal exposure, the number of opportunities for a regression, derived from consecutive valid fixations assigned to score notes. An additional exploratory analysis examined a temporal-compensation account. A complete overview of the supplementary models is provided in Supplementary Table S1a.

1. Rate-based robustness models

The rate-based robustness models retained regressive-fixation count as the outcome but included log-transformed performance duration as an offset. M4Rate corresponded to Model 4 with performance duration treated as exposure rather than as a conventional predictor, whereas SensFixDRate additionally controlled for mean fixation duration. These models tested whether the EHST × R-accuracy interaction remained when regressive fixations were modeled relative to execution time.

2. Regression-probability robustness models

The regression-probability robustness models used binomial mixed-effects models with a logit link. For each participant–phrase observation, regressions were modeled as successes relative to the total number of opportunities for a regression derived from consecutive valid fixations assigned to score notes. SensRegProb retained the fixed-effect structure of Model 4, and SensRegProbFixD additionally controlled for mean fixation duration. These models tested whether the predictors were associated with the probability that a valid fixation event was regressive, rather than only with the absolute number of regressive fixations.

3. Exploratory temporal-compensation analysis

A separate emergent exploratory linear mixed-effects analysis tested whether shorter fixation durations attenuated the association between producing more regressive fixations and taking longer to perform a phrase. The outcome was the log ratio between observed and MIDI-reference phrase duration. Regressive-fixation count and mean fixation duration were decomposed into within-participant and between-participant components, with the within-participant interaction providing the key test of temporal compensation.

## 3. Results

### 3.1. Descriptive Statistics

Descriptive statistics for the variables included in the random forest and mixed-effects analyses are presented in Table 2. Descriptive statistics for several additional variables considered in the random forest analyses but not retained in the reduced predictor set are provided in Supplementary Table S2a,b, and correlations among the principal participant-level means are reported in Supplementary Table S3.

**Figure 2:**
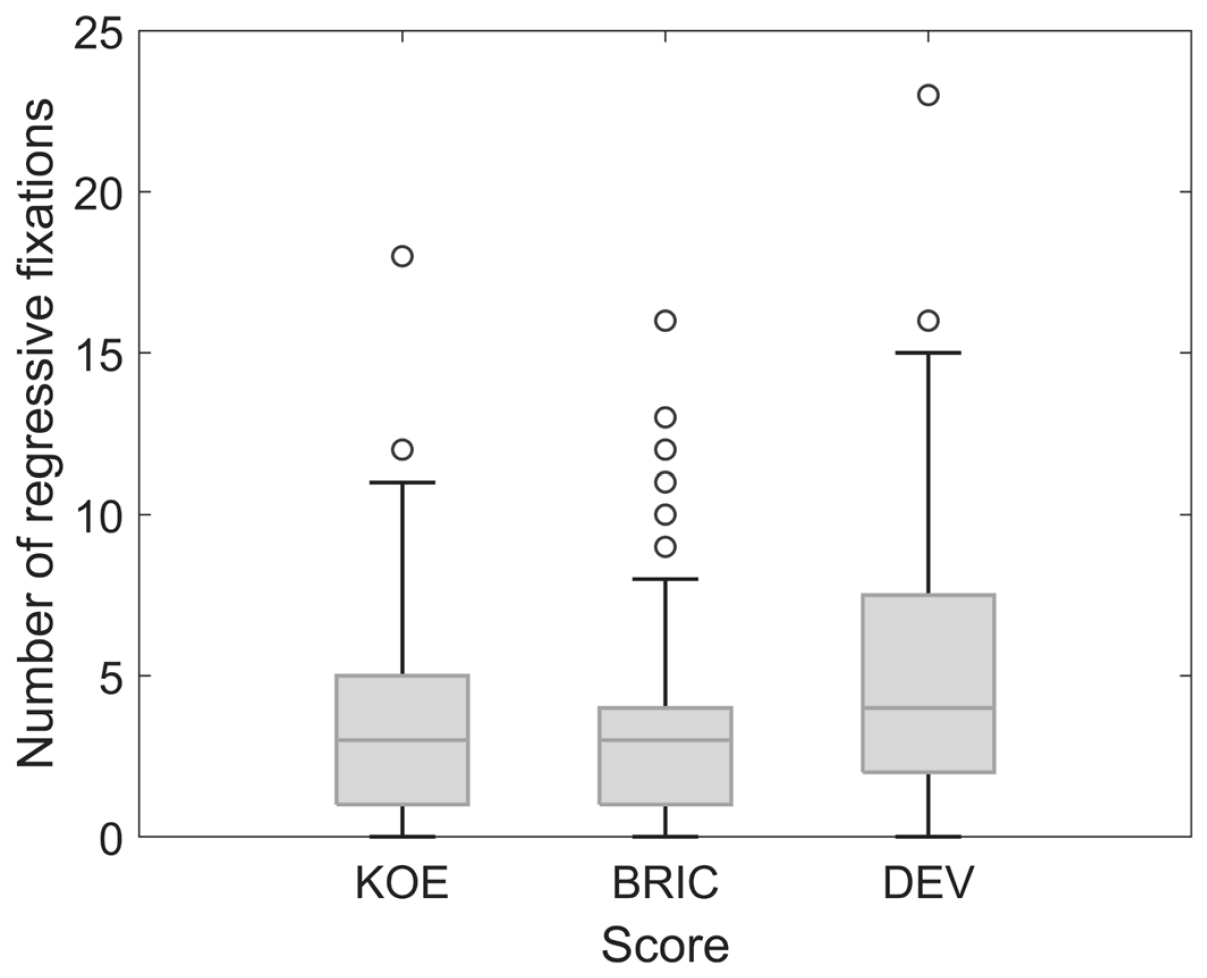
Distribution of regressive-fixation counts by score. Values represent participant-by-phrase observations; each value corresponds to the number of regressive fixations produced by one participant in one phrase. Boxes show the interquartile range, horizontal lines indicate medians, whiskers extend to the most extreme non-outlying observations, and circles indicate observations beyond 1.5 × IQR.

Regarding regressive fixations and related measures, 1566 of 16,132 opportunities for regression, between consecutive valid fixations assigned to score notes, were classified as regressive, yielding a weighted proportion of 9.71%. Regressive-fixation counts were highest in DEV, followed by KOE and BRIC (see Table 2 and Figure 2). Figure 2 further shows generally low-to-moderate counts across scores, with medians around three fixations in KOE and BRIC and slightly higher values in DEV. DEV showed the widest distribution and the highest extreme value, although high outlying observations were present in all three scores. Mean regression size differed only modestly across scores. Regression-size distributions were strongly right-skewed across all three scores, with one-note regressions constituting the largest category in BRIC, KOE, and DEV (see Supplementary Figure S2). Phrase-level regression proportions showed the same pattern, with DEV exhibiting the highest values, followed by KOE and BRIC (see Supplementary Table S2a,b).

### 3.2. Random Forest Models

A random forest regression model was fitted to predict the number of regressive fixations during sight-reading of the three musical scores. The analysis included 409 complete participant-by-phrase observations, corresponding to four phrases in KOE, seven in BRIC, and four in DEV. Predictive performance was evaluated using participant-grouped cross-validation, ensuring that observations from the same musician were not split across training and test folds. Across five participant-grouped cross-validation repetitions, the focal model achieved a mean out-of-fold R^2^ of 0.60 (*SD* = 0.02), a mean RMSE of 2.14 fixations (*SD* = 0.06), and a mean MAE of 1.61 fixations (*SD* = 0.03).

Permutation-based variable importance identified fixation count as the strongest predictor of regressive-fixation count (Table 3). This result was interpreted cautiously because regressive fixations are a subset of all fixations, such that fixation count likely indexed the opportunity for regressions to occur rather than an independent explanatory mechanism. Consistent with this interpretation, jointly permuting fixation count and mean fixation duration produced the largest loss in predictive performance, reducing out-of-fold R^2^ from 0.60 to −0.07 (ΔR^2^ = 0.67). Permuting fixation count alone also produced a large reduction in performance (ΔR^2^ = 0.518), whereas the temporal component of the predictor set, comprising performance duration, rhythmic accuracy, and EHST, produced a smaller but still noticeable reduction (ΔR^2^ = 0.13). When fixation count was excluded from the predictor set and the random forest was refitted, predictive performance decreased to R^2^ = 0.49, indicating that FixC captured a considerable opportunity-related component of the raw regression count, although the remaining temporal and oculomotor predictors retained meaningful predictive information. For this reason, fixation count was not included as a focal predictor in the subsequent explanatory mixed-effects count models; differences in the number of opportunities for a regression were instead addressed in the regression-probability robustness models.

An expanded random forest model additionally including flute-playing experience and breathing rate reduced out-of-fold predictive performance from R^2^ = 0.60 to R^2^ = 0.57, and these added variables were among the lowest-ranked predictors. Accordingly, the subsequent mixed-effects analyses focused on temporal anticipation, performance duration, rhythmic accuracy, and fixation duration as explanatory predictors of regressive-fixation count, while retaining Score as a prespecified control factor.

### 3.3. Linear Mixed Models

#### 3.3.1. Primary Count-Models

Results from the progressive Poisson mixed-effects models of regressive-fixation count are summarized in Table 4 and Table 5. Despite the strong participant-level correlation between performance duration and rhythmic accuracy, variance inflation factors calculated for the phrase-level fixed-effect predictors ranged from 1.21 to 3.46, with the highest value observed for performance duration. These values did not suggest problematic multicollinearity (see Supplementary Table S4).

##### Model 1

Temporal and note-based anticipation. In Model 1, EHST was strongly and positively associated with the number of regressive fixations, β = 0.691, 95% CI [0.554, 0.828], *p* < 0.001. Thus, fixations occurring earlier relative to the corresponding musical execution were associated with a larger number of regressive fixations. EHSN showed a small but statistically significant negative association, β = −0.190, 95% CI [−0.343, −0.037], *p* = 0.015.

##### Model 2

Control for performance duration. Performance duration was introduced in Model 2 to account for differences in the time available for oculomotor activity during musical performance. The inclusion of performance duration in M2 substantially attenuated the EHST coefficient, from β = 0.691 (*p* < 0.001) in M1 to β = 0.270, 95% CI [0.094, 0.446], *p* = 0.003, in M2. This approximately 61% attenuation corresponded to a decrease in exp(β) from 2.00 to 1.31, suggesting that performance duration accounted for a substantial part of the unadjusted association. Despite this attenuation, EHST remained significantly associated with regression count after adjustment for performance duration. Performance duration was strongly and positively associated with the number of regressive fixations, β = 0.063, 95% CI [0.047, 0.079], *p* < 0.001. After controlling for performance duration, EHSN changed from a small negative coefficient to a significant positive coefficient, β = 0.237, 95% CI [0.049, 0.425], *p* = 0.013. This sign reversal is consistent with suppression or shared variance involving performance duration, indicating that the association between note-based anticipation and regressive fixations was not stable across model specifications.

##### Model 3

Rhythmic accuracy did not show an independent main effect, β = 0.154, 95% CI [−0.573, 0.881], *p* = 0.678. EHST, EHSN, and performance duration remained significant after rhythmic accuracy was added to the model: EHST, β = 0.271, 95% CI [0.095, 0.447], *p* = 0.003; EHSN, β = 0.243, 95% CI [0.053, 0.433], *p* = 0.013; and performance duration, β = 0.065, 95% CI [0.045, 0.085], *p* < 0.001. The absence of a main rhythmic-accuracy effect indicates that more accurate performances did not simply contain more or fewer regressive fixations after accounting for temporal anticipation, note-based anticipation, and performance duration.

##### Model 4

**Figure 3:**
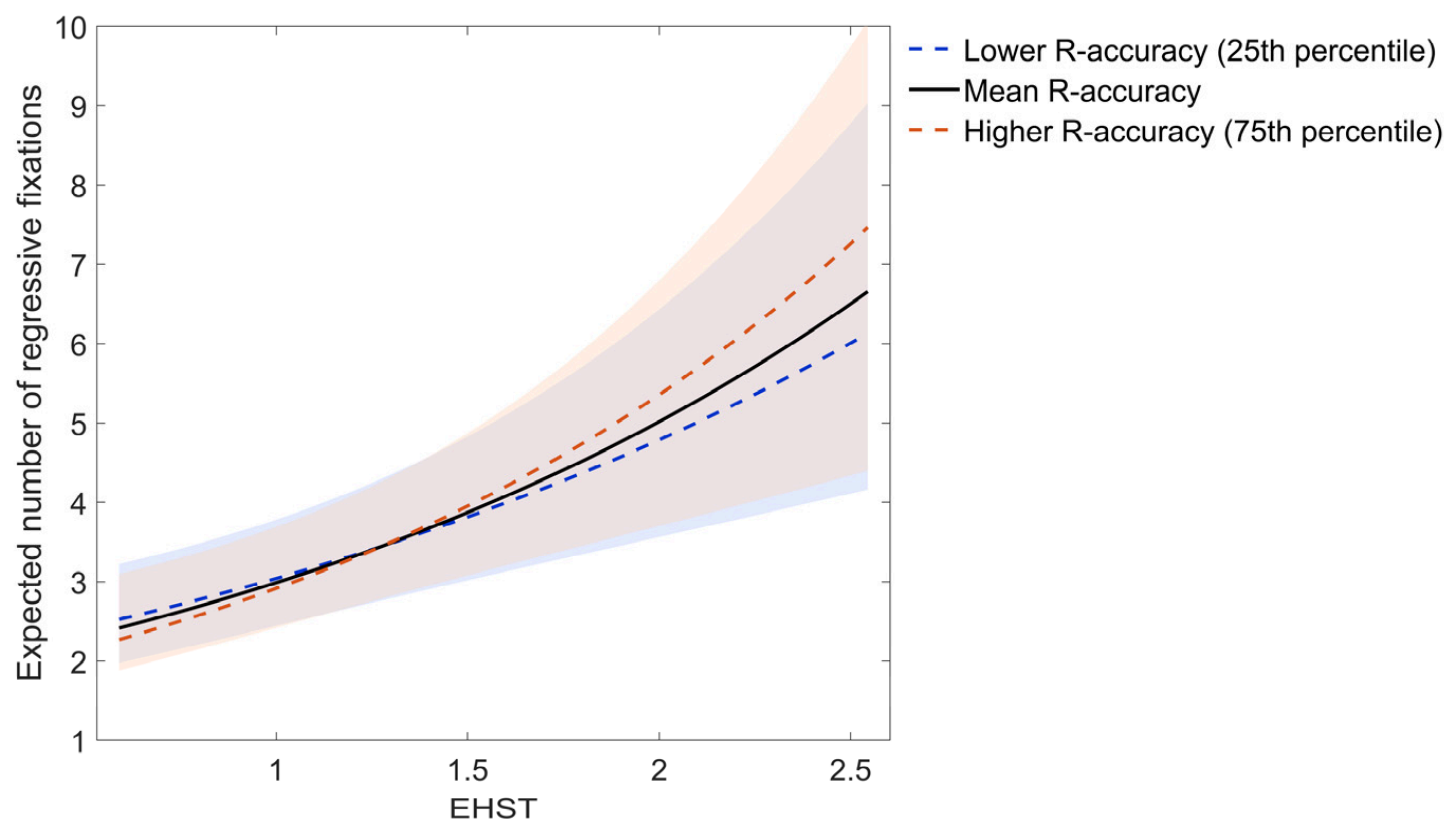
Model-based expected number of regressive fixations as a function of EHST and rhythmic accuracy. The lower, mean, and higher rhythmic-accuracy lines represent expected values evaluated at the 25th percentile, mean, and 75th percentile of the observed rhythmic-accuracy distribution, respectively. Shaded areas indicate 95% confidence intervals. Expected numbers of regressive fixations were derived from the main Poisson mixed-effects model (M4). EHST = time-based eye–hand span (s); R-accuracy = rhythmic accuracy.

Moderation by rhythmic accuracy. Model 4 tested whether rhythmic accuracy modified the relationship between temporal anticipation and regressive fixations. The EHST × rhythmic accuracy interaction was positive and significant, β = 1.062, 95% CI [0.282, 1.842], *p* = 0.008. This positive interaction indicates that the association between EHST and regressive fixations became stronger as rhythmic accuracy increased (see Figure 3). Simple-slope analyses further showed that EHST was positively associated with regressive-fixation count at the 25th percentile of rhythmic accuracy, β = 0.454, 95% CI [0.233, 0.675], *p* < 0.001; at mean rhythmic accuracy, β = 0.518, 95% CI [0.267, 0.770], *p* < 0.001; and at the 75th percentile, β = 0.609, 95% CI [0.307, 0.910], *p* < 0.001. Thus, the association between EHST and regressive-fixation count was present across the examined range of rhythmic accuracy but was stronger at higher rhythmic accuracy. The corresponding model-based expected number of regressive fixations, with 95% confidence intervals are shown in Figure 3. Performance duration also remained positively associated with regressive fixations, β = 0.061, 95% CI [0.041, 0.081], *p* < 0.001. The conditional effect of rhythmic accuracy at mean EHST was not significant, β = −0.111, 95% CI [−0.873, 0.651], *p* = 0.775, and EHSN was also not significant, β = 0.139, 95% CI [−0.065, 0.343], *p* = 0.184. The non-significant omnibus Score effect suggests that the EHST × R-accuracy interaction was not driven by simple mean differences among BRIC, DEV, and KOE, although score–phrase variability remained present.

Model diagnostics provided no evidence of overdispersion or substantial excess zero inflation. For the primary M4 model, the Pearson dispersion ratio was 0.861, with 40 observed zero-count observations compared with 37.51 expected under the fitted Poisson model. For the regression-size sensitivity model excluding one-note regressions, the corresponding dispersion ratio was 0.763, with 120 observed zeros compared with 120.43 expected zeros.

The simulation-based sensitivity analysis for Model 4 yielded an estimated detection probability of 76.2% for the EHST × rhythmic-accuracy interaction at α = 0.05. Across the 500 simulated datasets, 99.6% of the recovered interaction estimates retained the positive direction of the observed effect.

An additional spatial-assignment sensitivity analysis showed that the focal M4 results were robust to imposing explicit maximum fixation-to-note distance thresholds. With a 1.0° threshold (1373 retained regressive-fixation events), both the EHST main effect (β = 0.623, 95% CI [0.354, 0.893], *p* < 0.001) and the EHST × rhythmic-accuracy interaction (β = 1.171, 95% CI [0.261, 2.082], *p* = 0.012) remained positive and significant. The same pattern was observed under the stricter 0.6° threshold (1101 retained events): EHST, β = 0.712, 95% CI [0.407, 1.017], *p* < 0.001; EHST × rhythmic accuracy, β = 1.249, 95% CI [0.170, 2.329], *p* = 0.023. Thus, the focal interaction remained significant under both spatial-assignment thresholds and was not dependent on retaining more spatially distant fixation-to-note assignments.

A further sensitivity analysis examined whether the focal M4 findings were driven primarily by one-note regressions. After excluding these regressions, the EHST main effect remained significant (β = 0.602, 95% CI [0.217, 0.988], *p* = 0.002). The EHST × rhythmic-accuracy interaction retained a comparable positive effect estimate (β = 1.198, 95% CI [−0.012, 2.408], *p* = 0.052) but was estimated with greater uncertainty and no longer reached the conventional significance threshold. Full fixed- and random-effect estimates for the spatial-assignment and regression-size sensitivity models are reported in Supplementary Table S8.

##### Results of Sensitivity Analysis Controlling for Fixation Duration (SensFixD)

The sensitivity model included mean fixation duration, which was one of the highest-ranked predictors in the exploratory random forest analysis. Fixation duration was strongly and negatively associated with the number of regressive fixations, β = −0.302 per 100 ms, 95% CI [−0.363, −0.241], *p* < 0.001. Thus, longer mean fixation durations were associated with fewer regressive fixations. Crucially, introducing fixation duration did not attenuate the central EHST × rhythmic-accuracy interaction, which remained significant and increased in magnitude, β = 1.220, 95% CI [0.459, 1.980], *p* = 0.002. EHST remained positively associated with regressive fixations, β = 0.495, 95% CI [0.254, 0.736], *p* < 0.001, and performance duration also remained positive, β = 0.060, 95% CI [0.042, 0.078], *p* < 0.001. The EHST and performance duration coefficients changed only modestly relative to Model 4, indicating stability after adjustment for general fixation duration. By contrast, EHSN was not associated with regressive fixations, β = −0.016, 95% CI [−0.214, 0.182], *p* = 0.872. This suggests that the EHSN association observed in the additive models was not robust once the EHST × rhythmic accuracy interaction and general fixation duration were considered. Descriptively, adding fixation duration reduced participant-level variance by approximately 60% and score–phrase variance by approximately 35% relative to Model 4. Thus, fixation duration appeared to account for a substantial portion of stable between-musician variability and some score–phrase variability, but it did not account for the interaction between temporal anticipation and rhythmic accuracy.

In summary, across the primary count models, the omnibus effect of Score was not significant, indicating that regressive-fixation counts did not differ reliably across BRIC, DEV, and KOE after accounting for the predictors included in each model. Random-effect estimates indicated residual variability at both the participant and score–phrase levels throughout the model sequence. The fixation-duration sensitivity model showed that mean fixation duration explained part of this residual variability, but the EHST × rhythmic accuracy interaction remained significant after controlling for fixation duration. Full fixed- and random-effect estimates are reported in Table 4 and Table 5.

#### 3.3.2. Results of Supplementary Robustness and Exploratory Analyses

##### Results of Rate-Based Robustness Models

As described in Section 2, supplementary rate-based versions of Model 4 and the fixation-duration sensitivity model were fitted to determine whether the observed effects remained after accounting for performance duration as temporal exposure. In these models, regressive-fixation count was retained as the outcome, but log-transformed performance duration was included as an offset rather than as a conventional predictor. M4Rate corresponded to Model 4, whereas SensFixDRate additionally controlled for mean fixation duration.

1. Results of Rate-based model with performance duration as an offset

In M4Rate, EHST remained positively associated with the expected regressive-fixation count per unit of performance time, β = 0.520, 95% CI [0.297, 0.743], *p* < 0.001. At mean rhythmic accuracy, a one-unit increase in EHST corresponded to an estimated incidence-rate ratio of 1.68, indicating a 68% higher expected count of regressive fixations per unit of performance time. More importantly, the EHST × rhythmic accuracy interaction remained significant, β = 0.898, 95% CI [0.130, 1.666], *p* = 0.023. Thus, the positive association between temporal anticipation and expected regressive-fixation count per unit of performance time became stronger as rhythmic accuracy increased. This result indicates that the interaction observed in the count model was not attributable solely to longer performances providing more time in which regressions could occur.

The conditional effect of rhythmic accuracy was negative, β = −0.936, 95% CI [−1.579, −0.293], *p* = 0.005. Because the model included an interaction and both predictors were centered, this coefficient represents the association between rhythmic accuracy and the expected regressive-fixation count per unit of performance time specifically at the mean level of EHST. It should therefore not be interpreted as evidence that greater rhythmic accuracy is generally associated with fewer regressive fixations. Instead, the combination of a negative conditional effect and a positive interaction suggests that, at average temporal anticipation, higher rhythmic accuracy was associated with lower expected counts of regressive fixations per unit of performance time, whereas this association became less negative as the temporal anticipation window expanded. EHSN showed a positive but nonsignificant association, β = 0.140, 95% CI [−0.027, 0.307], *p* = 0.101.

2. Results of Rate-based model controlling for fixation duration

The main findings were preserved when mean fixation duration was added in SensFixDRate. EHST remained strongly and positively associated with regressive-fixation count per unit of performance time, β = 0.482, 95% CI [0.272, 0.692], *p* < 0.001, corresponding to an incidence-rate ratio of approximately 1.62 at mean rhythmic accuracy. The EHST × rhythmic accuracy interaction also remained significant and increased modestly in magnitude relative to M4Rate, β = 1.056, 95% CI [0.305, 1.807], *p* = 0.006. Thus, fixation duration did not account for the stronger association between temporal anticipation and expected regressive-fixation count per unit of performance time at higher levels of rhythmic accuracy.

Mean fixation duration was strongly and negatively associated with regressive fixations, β = −0.305 per 100 ms, 95% CI [−0.364, −0.246], *p* < 0.001. Expressed per 100 ms, this corresponds to an estimated rate ratio of approximately 0.74, or about 26% fewer regressive fixations per unit of performance time. Shorter fixation durations were therefore associated with more regressive fixations per unit of performance time. The conditional effect of R-accuracy remained negative, β = −0.870, 95% CI [−1.482, −0.258], *p* = 0.006, whereas EHSN was not associated with regressive-fixation count per unit of performance time after fixation duration was controlled, β = −0.005, 95% CI [−0.170, 0.160], *p* = 0.956. This attenuation suggests that the association between EHSN and regressive fixations observed in M4Rate may partly reflect variance associated with mean fixation duration rather than an independent contribution of note-based anticipation.

Across both rate-based models, the omnibus effect of Score was not significant, indicating that expected regressive-fixation counts per unit of performance time did not differ reliably across BRIC, DEV, and KOE after accounting for the predictors included in each model. Random-effect estimates indicated residual variability at both the participant and score–phrase levels. Descriptively, adding fixation duration reduced the participant variance by approximately 61% and the score–phrase variance by approximately 44%. Mean fixation duration therefore appeared to capture a substantial portion of residual participant-level variability and part of the residual score–phrase variability in time-adjusted regressive-fixation count. Nevertheless, it did not explain the interaction between temporal anticipation and rhythmic accuracy. Full fixed- and random-effect estimates are reported in Supplementary Table S5.

In summary, the rate-based analyses closely reproduced the central findings of the corresponding count models. The coefficient for the EHST × rhythmic accuracy interaction decreased from 1.062 in the principal count model to 0.898 in M4Rate, an attenuation of approximately 15%, while remaining significant. In the fixation-duration sensitivity analysis, the coefficient decreased from 1.220 in the count model to 1.056 in SensFixDRate, an attenuation of approximately 13%, and again remained significant. Consequently, the modest attenuation suggests that differences in performance duration explained only part of the interaction. Temporal anticipation was associated not only with the total number of regressions, but also with the expected count of regressive fixations per unit of performance time, particularly in rhythmically accurate performances.

##### Results of Regression-Probability Robustness Models

A second set of robustness models examined whether the main count-model interaction remained after accounting for differences in the number of opportunities for a regression. In the primary regression-probability model (SensRegProb), the EHST × rhythmic accuracy interaction remained significant, β = 1.052, 95% CI [0.230, 1.874], *p* = 0.012. This suggests that the interaction observed for regressive-fixation count was not explained solely by differences in the number of opportunities for a regression. Thus, the positive coupling between temporal anticipation and regressive eye movements was also present when the outcome was expressed as regression probability rather than as a raw count. At the mean level of R-accuracy, higher EHST was associated with higher regression probability, β = 0.496, 95% CI [0.235, 0.757], *p* < 0.001. This indicates that greater temporal anticipation was associated not only with more regressive fixations overall, but also with a higher probability of regression.

The interaction also remained significant after mean fixation duration was added in SensRegProbFixD, β = 1.157, 95% CI [0.340, 1.974], *p* = 0.006. In this model, FixD was negatively associated with regression probability, β = −0.143 per 100 ms, 95% CI [−0.206, −0.080], *p* < 0.001, indicating that longer mean fixation durations were associated with lower odds of regression. Together, these results suggest that the EHST × rhythmic accuracy interaction was not explained solely by differences in the number of opportunities for a regression or by general fixation duration. Full model estimates are reported in Supplementary Table S6, and the EHST × rhythmic-accuracy interaction from the primary regression-probability model (SensRegProb) is illustrated in Supplementary Figure S1.

##### Results of Exploratory Temporal-Compensation Analysis

A separate exploratory supplementary analysis addressed the emergent question of whether shorter fixation durations attenuated the association between regressive-fixation counts and longer phrase-level performance duration. Within participants, increases in regressive-fixation count were associated with longer relative performance duration compared with the MIDI reference, *b* = 0.022, 95% CI [0.015, 0.028], *p* < 0.001. Increases in mean fixation duration were also independently associated with longer relative performance duration, *b* = 0.033 per 100 ms, 95% CI [0.019, 0.048], *p* < 0.001. However, the within-participant interaction between regressive-fixation count and mean fixation duration was not significant, *b* = −0.002, 95% CI [−0.008, 0.003], *p* = 0.404. The likelihood-ratio comparison likewise did not favor the interaction model, LR χ^2^(1) = 0.67, *p* = 0.412. This indicates that adding the RegC_WP × FixD100_WP interaction did not significantly improve model fit over the corresponding additive model. Thus, the analysis provided no evidence that shorter fixation durations compensated for the additional performance time associated with exhibiting more regressions. Instead, regressive-fixation count and mean fixation duration showed separate positive associations with relative performance duration. Full model estimates are reported in Supplementary Table S7.

## 4. Discussion

The present study examined regressive fixations in relation to anticipatory mechanisms and the temporal organization of sight-reading in advanced flutists performing three musical scores with regular and irregular metrical structures. Across the mixed-effects analyses, the temporal component of sight-reading emerged as the most consistent correlate of regressive-fixation count, with time-based anticipation showing the most robust association whereas note-based anticipation showed a non-equivalent pattern of association. With respect to H2, the findings indicate that temporal and note-based anticipation were differentially associated with regressive-fixation count, supporting the view that they reflect distinguishable components of anticipatory processing. Performance duration also contributed substantially to the observed variability. Most importantly, rhythmic accuracy moderated the relationship between EHST and regressive-fixation count: the positive association was stronger in performances that reproduced the notated temporal structure more accurately. This interaction remained significant in the rate models and after controlling for mean fixation duration, providing broad support for the rhythmic-accuracy moderation hypothesis (H3). The random forest analysis added predictive validation to the study by showing moderate-to-strong out-of-sample prediction of regressive-fixation count in held-out participants, R^2^ = 0.60, thereby supporting H1. Together, these findings suggest that regressive fixations may contribute to integrative mechanisms through which anticipatory information is continuously coordinated with the temporal demands of ongoing performance.

### 4.1. Regressive Fixations Indexing Temporal Processing in Music Reading

The results indicate that regressive fixations were systematically associated with temporal anticipation and performance duration. A larger EHST indicates that musical information was inspected earlier relative to its execution. Across both count and rate models, a larger temporal eye–hand span was associated with more regressions. Anticipation and regression should therefore not be regarded as mutually exclusive behaviors. A larger EHST value implies that visually acquired information must remain available for longer before being translated into motor and acoustic output. During this interval, regressive fixations may allow musicians to revisit previously inspected notation while anticipatory information about upcoming musical material is retained. This interpretation is compatible with a temporally maintained perception–action buffer. Within this account, such regressions may be associated with the process of verification or reintegration of rhythmic groupings, melodic relations, or note–action mappings, without implying that every regression serves the same function. This account is consistent with the Information Gathering Framework, which treats progressive and regressive eye movements as responses to changing confidence in currently available information rather than as fixed markers of successful or unsuccessful reading [42].

The interaction with rhythmic accuracy further qualifies this interpretation. Performances with greater rhythmic accuracy were not characterized by a uniformly higher regressive-fixation count. Rather, the association between EHST and regressive-fixation count became stronger as rhythmic accuracy increased. This pattern can be interpreted within the predictive coding of music framework, according to which temporal expectations are generated from internal models of rhythmic and metrical structure and continuously compared with unfolding musical input and action [34,36]. Enhanced rhythmic accuracy could reflect more stable or precise temporal predictions. Under these conditions, a longer anticipatory window could coexist with selective returns to previously inspected notation.

A complementary perspective comes from task-control accounts of gaze, which propose that eye movements are organized around task-relevant events and upcoming actions [78,79]. Although these accounts do not address regressive fixations directly, they provide a broader framework in which the role of a regression may depend on its temporal coordination with anticipatory processes and motor execution. The present findings therefore support an account based on coordination among anticipatory processes, oculomotor behavior, and action rather than on the number of regressive fixations alone. This interpretation also aligns with the concept of context-dependent visuomotor flexibility in music reading, whereby oculomotor behavior is adjusted to local notational demands rather than following a fixed pattern [17]. Accordingly, similar regressions may occur under different processing conditions, potentially accompanying the integration of upcoming musical information in some cases, reflecting unresolved processing demands in others, or arising from oculomotor correction. This view is consistent with previous evidence that eye-movement patterns in music reading, including regressions, vary with expertise, notational organization, reading stage, and local processing demands [5,23,43,44,46,80].

The regression-size sensitivity analysis further indicated that the EHST association was not driven solely by one-note regressions. Notably, the EHST × rhythmic-accuracy interaction retained the same positive direction and a comparable, slightly larger effect estimate after one-note regressions were excluded, although with greater uncertainty, warranting greater caution in interpreting the moderation effect.

### 4.2. Temporal Control of Regressive Fixations: Performance and Fixation Dynamics

Performance duration formed a stable component of the findings. Longer performances contained more regressive fixations after adjustment for anticipation and the remaining covariates. The substantial attenuation of the EHST coefficient after performance duration was introduced (from β = 0.691 in M1 to β = 0.270 in M2) indicates that performance duration accounts for part of the association between temporal anticipation and regression count, probably because longer performances provide more time for regressions to occur. However, EHST remained significantly associated with regressions when performance duration was incorporated as an offset (see Supplementary Table S5) and in binomial models estimating regression probability relative to the number of valid consecutive fixation comparisons (see Supplementary Table S6), including after adjustment for mean fixation duration. Taken together, these findings suggest that performance duration contributes to, but does not fully account for, the association between temporal anticipation and regressions.

Mean fixation duration provided an additional perspective on this temporal organization. Shorter mean fixations were associated with more regressions in both the count and exposure-adjusted models. This association suggests that briefer fixations and more frequent regressions to previously inspected notation may coexist as partly distinct components of oculomotor behavior during performance. Mean fixation duration also contributed to the predictive analysis and was therefore included in a sensitivity model. The persistence of the EHST × rhythmic-accuracy interaction after controlling for mean fixation duration indicated that the central relationship was not attributable to general differences in fixation duration.

This pattern can be considered in relation to the Information Gathering Framework, which treats fixation prolongation and the initiation of a regression as distinct responses to changing information needs and confidence during reading [42]. In verbal reading, fixations immediately preceding regressions were generally shorter than those preceding progressive saccades, although the framework distinguishes regressions associated with integration difficulties from those arising from insufficient evidence. The present result is not directly equivalent, because mean fixation duration was calculated across each musical phrase rather than specifically for fixations preceding regressions. Nevertheless, the parallel suggests that shorter mean fixation durations and more frequent regressions may represent coordinated but partly independent adjustments to processing demands, rather than a simple compensatory mechanism in which shorter mean fixation durations offset the temporal cost associated with additional regressions.

This distinction motivated the exploratory supplementary analysis of a more specific temporal-compensation account (Supplementary Table S7). It tested whether the association between regressive-fixation count and relative performance duration was attenuated in phrases in which mean fixation durations were shorter than usual for the same musician. Such an interaction would be consistent with performers partly limiting the temporal cost of additional regressions through shorter mean fixation durations. However, the analysis did not provide robust support for this interaction. The results therefore did not support the proposed mechanism whereby shorter mean fixation durations compensate for the temporal cost associated with additional regressions. Moreover, because regressive-fixation count, regressive-fixation duration, and performance duration may influence one another, this observational analysis cannot establish causal relationships among these variables.

In the sensitivity count model, adding mean fixation duration reduced participant and score–phrase variance by approximately 60% and 35%, respectively. These descriptive reductions suggest that mean fixation duration captured part of the residual musician- and phrase-level variability. However, the reductions should not be interpreted as total variance explained or as evidence that fixation duration directly measures expertise. Previous findings nevertheless suggest that fixation number and duration may reflect partly distinct adaptations to musical demands. Expertise has frequently been associated with shorter fixation durations, while experienced musicians also adapt fixation duration, saccade amplitude, and visuomotor lead more flexibly to local complexity [8,17]. Structurally demanding sections may simultaneously require additional inspections and changes in fixation duration, as reported in relation to musical complexity and thematic development [15,51]. Thus, an increase in the number of inspections need not be accompanied by a proportional increase in the duration of each fixation. This distinction helps contextualize the findings of Drai-Zerbib and Baccino [5], who classified musical-reading expertise partly on the basis of total fixation count and found that the resulting groups differed in fixation duration and regressive-fixation measures. Musicians classified as less expert produced more regressive fixations, whereas those classified as expert showed fewer and shorter fixations. The effects of phrasing marks were also expertise-dependent, suggesting that less expert readers relied more strongly on written phrasing cues, while expert readers were less affected by their presence or absence.

The present findings therefore suggest that fixation duration and regressions reflect related but partly separable adjustments. Shorter mean fixation durations coexisted with more regressions, but shorter mean fixation durations did not attenuate the association between additional regressions and relative performance duration. The temporal organization of performance may consequently depend less on shortening individual regressive-fixation durations than on the relationship between anticipation and rhythmic execution, as reflected in the EHST × rhythmic-accuracy interaction.

Secondary physiological indicators were interpreted cautiously. Blink count ranked below the main temporal and fixation-duration predictors but above Score in the focal random forest, suggesting some predictive information beyond score identity. This finding is compatible with evidence linking spontaneous blinking to attentional fluctuations, response completion, and boundaries in continuous information processing [81,82,83], and with related flute research in which blink dynamics and breathing patterns formed part of a physiological-regulation dimension [18]. Consistent with this pattern, an expanded random forest model including breathing rate and flute-playing experience reduced out-of-fold prediction from R^2^ = 0.60 to R^2^ = 0.57, and these variables were among the lowest-ranked predictors. Thus, blink and respiratory measures are best viewed here as secondary indicators of the broader regulatory context of performance, rather than as primary explanations of regressive-fixation count.

### 4.3. Score-Level and Phrase-Level Variability

Score effects were not robust in the revised mixed-effects analyses. Neither the omnibus effect of score nor individual contrasts remained significant after accounting for anticipation, performance duration, rhythmic accuracy, and fixation duration. Although this finding was unexpected given the irregular meter of DEV relative to the regular meters of BRIC and KOE, it should not be interpreted as evidence that metric irregularity has no influence on regressions, since irregular meter was represented by a single score and therefore could not be disentangled from its other musical characteristics. This pattern is nevertheless consistent with previous studies showing that differences in musical material do not necessarily translate into global differences in regressive eye-movement patterns. Wurtz et al. [12] observed only a nonsignificant tendency toward more regressions in a more complex excerpt, whereas Cara and Gomez Vera [46] found, during silent score reading, that tonal and contemporary music differed in the distribution of regressive fixations across intra- and inter-phrase processing rather than in a single global measure of eye-movement behavior.

Musical enculturation may also have contributed to these findings. Li et al. [6] demonstrated that notation-specific experience influences regressions, while Haumann et al. [84] showed that musical enculturation shapes expectations concerning recurrent metrical structures. Because all participants were trained in conventional staff notation, variability associated with notational decoding was likely limited. By contrast, the irregular meter of DEV was relatively uncommon within the repertoire to which participants had been predominantly exposed during their training and may therefore have challenged culturally acquired metrical expectations. However, any influence of metrical familiarity was not sufficiently strong to produce a robust score-level effect on regressions once anticipation, performance duration, rhythmic accuracy, and fixation duration were taken into account. This suggests that metrical structure effects are more likely to emerge in specific oculomotor components rather than as global changes in the overall occurrence of regressions.

Finally, the non-zero score–phrase variance component indicated substantial phrase-level variation beyond the fixed predictors, suggesting that regressions were more strongly associated with variation among phrases than among scores. This interpretation is consistent with evidence that musical structure may differentially influence temporal and note-based anticipation [63] and with the present finding that rhythmic accuracy moderated the relationship between EHST and regressions across complementary outcome measures. Methodologically, these findings support modeling score phrase as a random grouping factor and reinforce the musical phrase as a meaningful unit for examining the temporal organization of gaze during sight-reading performance.

### 4.4. Cross-Domain Implications for Integrative Processing

The purpose of relating the present findings to verbal reading is not to claim empirical equivalence between domains, but to identify variables that may help characterize the integrative role of regressive eye movements. Whereas verbal comprehension is supported by well-established theoretical frameworks, musical comprehension lacks a single operational definition. In this study, rhythmic accuracy was therefore treated as a performance-based index of temporal integration, reflecting the extent to which score-derived duration relations were organized into a coherent and executable temporal structure. It does not represent a complete measure of musical comprehension, but an operational indicator of temporal-musical integration within the present design.

This interpretation aligns with construction–integration accounts of comprehension, in which information is continuously integrated into coherent representations extending beyond local elements [85,86]. It is also consistent with the RI-Val model, which conceptualizes comprehension as the dynamic interplay of activation, integration, and validation processes [87,88]. Predictive-coding frameworks in music further propose that metrical and rhythmic expectations are continuously updated against unfolding auditory–motor input [34,36]. Within this perspective, rhythmic accuracy can be treated here as an observable performance outcome reflecting the integration of predictive processes, structural knowledge, and performance execution. The EHST × rhythmic-accuracy interaction further suggests that the relationship between temporal anticipation and regressions emerges from the dynamic coordination of these processes during performance.

Evidence from verbal reading indicates that regressions support reanalysis, integration, and coherence maintenance, and are sensitive to informational demands and temporal coordination constraints [89,90]. The eye–voice span literature provides complementary evidence that temporal coordination is also relevant. In oral reading, a larger spatial eye–voice span has been associated with an increased probability of regressions and refixations [28]. In a related but methodologically distinct domain, temporal eye–voice span has also been positively associated with regression frequency during serial naming [29]. Computational models of reading offer complementary accounts of how forward encoding and regressions can emerge from different processing architectures, including the serial E-Z Reader model, the distributed SWIFT model, and the memory-integrated SEAM framework [91,92,93]. Together, they illustrate that encoding, integration, and reprocessing of previously encountered information can be dynamically coordinated over time during reading.

Finally, the present findings show that the relationship between temporal anticipation and regressive fixations is modulated by rhythmic accuracy. More broadly, the number of regressions alone appears insufficient to characterize their functional significance, as regressions may reflect correction, verification, or boundary-related integration. The observed association with EHST suggests that part of this behavior is linked to anticipatory temporal processing during music sight-reading. Although these models do not establish causal relationships and therefore do not demonstrate that regressive eye movements improve rhythmic accuracy, they do show that regressive eye movements become more strongly associated with temporal anticipation under conditions of greater rhythmic accuracy. These findings motivate further investigation of whether the functional role of regressive eye movements depends on their interaction with anticipatory processing across different sequential reading tasks.

### 4.5. Limitations and Future Directions

The findings should be interpreted in light of several limitations. First, regressive fixations were analyzed collectively at the musical-phrase level. Because the scores differed in layout, phrase structure, and note density, the study could not apply a common objective criterion for distinguishing local from global processing or determine the function of individual regressions. Event- or region-level analyses would be required to relate each regression to its immediate notational context. Note-level fixation assignment warrants some caution in visually dense regions, particularly in BRIC, where median adjacent-note spacing was closer to median calibration accuracy. However, the focal M4 findings remained robust when fixation-to-note assignments were restricted using explicit spatial-distance thresholds. The EHST main effect also remained significant after excluding one-note regressions, whereas the EHST × rhythmic-accuracy interaction retained a comparable positive effect estimate but was less precisely estimated.

Second, the scores differed metrically, with two regularly metered scores and one irregularly metered score. Although score was included as a control factor, metrical organization could not be separated from the other characteristics of each composition. In addition, DEV was consistently presented first, whereas the order of BRIC and KOE was counterbalanced across participants. Consequently, score identity and presentation position were partially confounded for DEV, and potential order-related effects, including learning or fatigue, cannot be completely ruled out. Execution tempo was also neither experimentally controlled nor included as an independent predictor. Performance duration captured the total elapsed time of phrase execution, whereas rhythmic accuracy quantified correspondence with a reference model defined at a particular tempo; consequently, execution speed and performance duration cannot be fully separated. Future studies should use matched materials, multiple examples of each metrical condition, and experimentally manipulated tempi.

Finally, rhythmic accuracy provides only an indirect indicator of temporal-musical integration. It captures the realized temporal organization of the score but cannot reveal the content, completeness, or coherence of the musician’s internal representation. Moreover, the EHST × rhythmic-accuracy interaction cannot establish that regressive fixations facilitate information reacquisition or improve rhythmic accuracy. The connections drawn with Kintsch’s construction–integration model, the RI-Val model, and predictive-coding accounts of music therefore remain conceptual. Future research could combine event-level regression classification with memory probes, structural judgments, differentiated performance-error measures, blink and respiratory timing, and controlled manipulations of musical structure.

## 5. Conclusions

The findings support a multiscale account in which mean fixation duration reflects fixation-level temporal dynamics, EHST indexes temporal anticipation during sight-reading, and performance duration captures total elapsed phrase time. Regressive-fixation count was most consistently associated with EHST, and this relationship became stronger as rhythmic accuracy increased. Crucially, this interaction remained significant when performance duration was treated as an exposure term and when mean fixation duration was included as a covariate. Thus, more accurate performances were not characterized by a general increase in regressions, but by a stronger relationship between regressive eye movements and temporal anticipation.

The random forest analysis complemented the mixed-effects results by identifying fixation count, mean fixation duration, and EHST as leading predictors. However, fixation count was interpreted cautiously because it likely reflected overall oculomotor activity and the number of available opportunities for regression derived from consecutive fixations, rather than an independent explanatory mechanism. Within this framework, mean fixation duration captured part of the variability across musicians and score phrases, with shorter mean fixation durations associated with higher regressive-fixation counts. However, supplementary analyses did not support the emergent hypothesis that shorter mean fixation durations offset the temporal cost associated with producing additional regressions. Mean fixation duration and regressive fixations appear to reflect related, but partly independent, adjustments in gaze organization during performance. Blink and breathing-rate measures contributed less to predictive performance, suggesting that regressions are more closely associated with oculomotor and temporal variables, although they may still be embedded within broader physiological regulation.

Although the scores included both regular and irregular metrical structures, the effect of score was not robust after accounting for the main predictors. The association between EHST, rhythmic accuracy, and regressive-fixation count was consistent across the three musical materials. However, because metrical irregularity was represented by a single score, the present design cannot isolate independent effects of metrical structure.

Importantly, the results do not indicate that regressions improve rhythmic accuracy, that extended anticipatory spans directly enhance performance, or that regressions are intentionally deployed. Rather, they suggest that the relationship between temporal anticipation and regressive eye movements is stronger when the notated temporal structure is performed more accurately. Regressive fixations may therefore be compatible with anticipatory processing and the integration of notational information that remains relevant during unfolding performance into ongoing execution. This interpretation extends functional parallels with eye–voice span and reading research by suggesting that the integrative role of regressions depends not only on the nature of the musical information being updated, but also on how temporal anticipation is aligned with the accuracy of ongoing performance execution.

## Figures and Tables

**Table 1:** Fixed and random-effects specifications of the primary count models and fixation-duration sensitivity model.

Model	Specification	Purpose
Model 1	RegC ~ EHST + EHSN + Score + (1|Participant) + (1|ScorePhrase)	Examines the associations of EHST and EHSN with regressive-fixation count.
Model 2	RegC ~ EHST + EHSN + PerfD + Score + (1|Participant) + (1|ScorePhrase)	Tests whether associations of EHST and EHSN with regressive-fixation count persist after controlling for performance duration.
Model 3	RegC ~ EHST + EHSN + PerfD + R-accuracy + Score + (1|Participant) + (1|ScorePhrase)	Examines whether rhythmic accuracy is additionally associated with regressive-fixation count after accounting for EHST, EHSN and performance duration.
Model 4	RegC ~ EHST * R-accuracy + EHSN + PerfD + Score + (1|Participant) + (1|ScorePhrase)	Tests whether rhythmic accuracy moderates the association between EHST and regressive-fixation count.
SensFixD	RegC ~ EHST * R-accuracy + EHSN + PerfD + FixD + Score + (1|Participant) + (1|ScorePhrase)	Tests the robustness of Model 4 after controlling for mean fixation duration.

Note. All models used regressive-fixation count as the outcome and were fitted using a Poisson distribution with a log link. EHST, EHSN, PerfD, R-accuracy, and FixD were grand-mean centered before model fitting. The asterisk (*) in model specifications denotes the inclusion of the corresponding main effects and their interaction. RegC = regressive-fixation count; EHST = time-based eye–hand span; EHSN = note-based eye–hand span; PerfD = phrase performance duration; R-accuracy = rhythmic accuracy; FixD = mean fixation duration. Score was a categorical factor with three levels, BRIC, DEV, and KOE, and was effects coded. Score–phrase identified each phrase within its corresponding musical score. Random intercepts were included for participant and score–phrase.

**Table 2:** Means, standard deviations, and 95% confidence intervals for performance, oculomotor, anticipatory, and respiratory measures across the three musical scores.

Measures	*Mean*			*SD*			95% CI					
							*LL*			*UL*		
	KOE	DEV	BRIC	KOE	DEV	BRIC	KOE	DEV	BRIC	KOE	DEV	BRIC
RegC	3.15	4.91	2.64	2.25	3.30	2.11	2.71	4.28	2.33	3.59	5.54	2.95
EHST	0.98	1.27	1.09	0.18	0.39	0.35	0.94	1.20	1.04	1.01	1.35	1.14
EHSN	2.05	1.77	2.20	0.36	0.29	0.53	1.98	1.72	2.12	2.12	1.83	2.27
PerfD	18.02	26.00	16.66	3.14	7.38	5.94	17.4	24.61	15.79	18.63	27.9	17.53
R-accuracy	1.01	1.46	0.73	0.13	0.43	0.11	0.99	1.38	0.72	1.04	1.54	0.75
FixD	437.22	497.79	473.26	102.31	137.18	134.88	417.51	471.50	453.64	456.92	524.09	492.88
BlinkC	6.64	11.09	5.19	4.34	7.62	3.67	5.79	9.61	4.64	7.49	12.56	5.73
BreathR	9.86	9.10	9.63	2.73	2.08	2.72	9.32	8.70	9.23	10.39	9.50	10.03

Note. CI = confidence interval; LL = lower confidence limit; UL = upper confidence limit; KOE = Koechlin excerpt; DEV = *Makedonsko devojče* (Hristovski) score; BRIC = Briccialdi excerpt; RegC = regressive-fixation count; EHST = time-based eye–hand span (s); EHSN = note-based eye–hand span; PerfD = phrase performance duration (s); R-accuracy = rhythmic accuracy on the original performed-to-reference duration-ratio scale; FixD = mean fixation duration (ms); BlinkC = blink count; BreathR = number of respiratory cycles per minute.

**Table 3:** Predictors included in the random forest model, ranked according to permutation-based variable importance.

Rank	Predictor	Importance
1	FixC	1.78
2	FixD	0.93
3	EHST	0.90
4	PerfD	0.59
5	R-accuracy	0.54
6	EHSN	0.39
7	BlinkC	0.34
8	Score	0.18

Note. Importance = mean permutation-based variable-importance score across five random-forest runs; FixC = fixation count; FixD = mean fixation duration; EHST = time-based eye–hand span; PerfD = phrase performance duration; R-accuracy = rhythmic accuracy; EHSN = note-based eye–hand span; BlinkC = blink count; Score = musical score (BRIC, DEV, or KOE).

**Table 4:** Fixed and random effects from the Poisson mixed-effects models predicting regressive-fixation counts.

	M1				M2				M3			
Fixed Effects	β	*SE*	*t*	*p*	β	*SE*	*t*	*p*	β	*SE*	*t*	*p*
Intercept	1.208	0.108	11.213	<0.001	1.160	0.104	11.198	<0.001	1.157	0.105	11.064	<0.001
EHST	0.691	0.070	9.892	<0.001	0.270	0.090	2.989	0.003	0.271	0.090	2.998	0.003
EHSN	−0.190	0.078	−2.440	0.015	0.237	0.096	2.483	0.013	0.243	0.097	2.502	0.013
Score BRIC	−0.214	0.104	−2.066	0.039	−0.109	0.089	−1.230	0.219	−0.099	0.094	−1.056	0.292
Score DEV	0.113	0.118	0.959	0.338	−0.036	0.102	−0.356	0.722	−0.032	0.105	−0.310	0.756
PerfD					0.063	0.008	7.728	<0.001	0.065	0.010	6.763	<0.001
R-accuracy									0.154	0.371	0.415	0.678
**Random Effects**	**M1**				**M2**				**M3**			
	**Variance**		***SD***		**Variance**		***SD***		**Variance**	***SD***		
Participant	0.148		0.385		0.172		0.415		0.172	0.415		
Score–phrase	0.077		0.277		0.051		0.225		0.053	0.231		
Omnibus Score	***F***	**df**		***p***	***F***	**df**	***p***		***F***	**df**		***p***
	2.135	2, 404		0.120	1.315	2, 403	0.270		0.909	2, 402		0.404

Note. β = unstandardized coefficient on the log scale; *SE* = standard error; *SD* = standard deviation; *t* = test statistic for individual fixed-effect coefficients; *F* = omnibus test statistic; df = degrees of freedom; *p* = *p*-value; EHST = time-based eye–hand span; EHSN = note-based eye–hand span; PerfD = phrase performance duration; R-accuracy = rhythmic accuracy. Models used a Poisson distribution with a log link to predict regressive-fixation count. Continuous predictors were grand-mean centered. Score was effect-coded using sum-to-zero contrasts across BRIC, DEV, and KOE; KOE was the implicit level and not a reference category. Random intercepts were included for participant and score–phrase. *N* = 409 participant–phrase observations.

**Table 5:** Fixed and random effects from the Poisson mixed-effects models predicting regressive-fixation counts: interaction and fixation-duration sensitivity models.

	M4				SensFixD			
Fixed Effects	β	*SE*	*t*	*p*	β	*SE*	*t*	*p*
Intercept	1.182	0.107	11.077	<0.001	1.151	0.080	14.471	<0.001
EHST	0.518	0.128	4.036	<0.001	0.495	0.123	4.041	<0.001
EHSN	0.139	0.104	1.330	0.184	−0.016	0.101	−0.162	0.872
Score BRIC	−0.143	0.099	−1.449	0.148	−0.101	0.085	−1.197	0.232
Score DEV	−0.049	0.108	−0.449	0.654	0.020	0.092	0.221	0.825
PerfD	0.061	0.010	6.173	<0.001	0.060	0.009	6.678	<0.001
R-accuracy	−0.111	0.389	−0.286	0.775	−0.004	0.368	−0.010	0.992
FixD (per 100 ms)					−0.302	0.031	−9.839	<0.001
EHST × R-accuracy	1.062	0.398	2.670	0.008	1.220	0.388	3.147	0.002
**Random Effects**	**M4**				**SensFixD**			
	**Variance**		***SD***		**Variance**		***SD***	
Participant	0.173		0.416		0.070		0.265	
Score–phrase	0.058		0.241		0.038		0.195	
Omnibus Score	***F***	**df**		***p***	***F***	**df**		***p***
	1.700	2, 401		0.184	0.752	2, 400		0.472

Note. β = unstandardized coefficient on the log scale; *SE* = standard error; *SD* = standard deviation; *t* = test statistic for individual fixed-effect coefficients; *F* = omnibus test statistic; df = degrees of freedom; *p* = *p*-value; EHST = time-based eye–hand span; EHSN = note-based eye–hand span; PerfD = phrase performance duration; FixD = mean fixation duration; R-accuracy = rhythmic accuracy. Models used a Poisson distribution with a log link to predict regressive-fixation count. Continuous predictors were grand-mean centered. Score was effect-coded using sum-to-zero contrasts across BRIC, DEV, and KOE; KOE was the implicit level and not a reference category. Random intercepts were included for participant and score–phrase. *N* = 409 participant–phrase observations.

## Data Availability

The data presented in this study are available on request from the corresponding author. The data are not publicly available due to privacy and confidentiality restrictions related to the participants.

## Supplementary Materials

The following supporting information can be downloaded at https://www.mdpi.com/article/10.3390/jemr19050102/s1, Figure S1: Predicted regression probability as a function of EHST and R-accuracy; Figure S2: Distribution of regression sizes by score; Table S1a: Overview of the supplementary robustness and exploratory analyses; Table S1b: Specifications of the spatial-assignment and regression-size sensitivity analyses; Table S2a: Means, standard deviations, and 95% confidence intervals for performance, oculomotor, anticipatory, and respiratory measures across the three musical scores; Table S2b: Medians and interquartile ranges for performance, oculomotor, anticipatory, and respiratory measures across the three musical scores; Table S3: Pearson correlations among participant-level mean oculomotor, respiratory, and performance measures during sight-reading; Table S4: Variance inflation factors for the fixed effects in the maximum additive model; Table S5: Poisson rate models with log-transformed performance duration as an offset; Table S6: Binomial mixed-effects models of regression probability; Table S7: Linear mixed-effects model for log-transformed relative performance duration: exploratory temporal-compensation analysis; Table S8: Poisson mixed-effects sensitivity models for spatial-assignment thresholds and regression-size exclusion.
