# Supplementary material for: Looking Back While Reading Ahead: Regressive Fixations and Temporal Anticipation in Music Sight-Reading
Source: J Eye Mov Res. 2026 Sep 15;19(5):102. doi: 10.3390/jemr19050102 (PMC13600049; doi:10.3390/jemr19050102)
Supplement: Supplementary file 1 [file jemr-19-00102-s001.zip › jemr-4516355-supplementary.pdf]

Journal of
Eye Movement Research

Looking Back While Reading Ahead: Regressive Fixations and Temporal Anticipation in Music Sight-Reading

Supplementary Material

Table S1a. Overview of the supplementary robustness and exploratory analyses.

Model	Distribution and link	Outcome	Specification	Exposure or denominator	Purpose
**Rate-based robustness models**					
M4Rate	Poisson; log	Regressive-fixation count	EHST * R-accuracy + EHSN + Score + (1 ∣ Participant) + (1 ∣ ScorePhrase)	Offset: log(PerfD)	Tests whether the EHST * R-accuracy interaction remains when regressive-fixation count is modeled relative to phrase performance duration.
SensFixDRate	Poisson; log	Regressive-fixation count	M4Rate + FixD	Offset: log(PerfD)	Tests whether the time-adjusted effects remain after controlling for mean fixation duration.
**Regression-probability robustness models**					
SensRegProb	Binomial; logit	Regression probability	EHST * R-accuracy + EHSN + PerfD + Score + (1 ∣ Participant) + (1 ∣ ScorePhrase)	Binomial size: total number of opportunities for a regression	Tests whether the findings remain when modeling regression probability instead of regressive-fixation count
SensRegProbFixD	Binomial; logit	Regression probability	SensRegProb + FixD	Binomial size: total number of opportunities for a regression	Tests whether the findings remain when modeling regression probability while controlling for mean fixation duration
**Exploratory temporal-compensation analysis**					
Compensation	Gaussian; identity	Log relative performance duration	RegC_WP * FixD100_WP + RegC_BP + FixD100_BP + EHST + EHSN + Score + (1 ∣ Participant) + (1 ∣ ScorePhrase)	Not applicable	Tests whether shorter-than-usual mean fixation durations attenuate the association between higher-than-usual regressive-fixation counts and longer relative performance duration.

Note. All mixed-effects models included random intercepts for participant and score-phrase. Continuous predictors were grand-mean centered before model fitting and were not standardized. The asterisk (*) denotes the inclusion of both main effects and their interaction. Score was effect-coded with three levels: BRIC, DEV, and KOE. In M4Rate and SensFixDRate, phrase performance duration was included as an offset, log(PerfD), rather than as a conventional predictor. In the regression-probability models, the number of regressions was modeled as successes out of the total number of valid consecutive fixation comparisons (i.e., opportunities for a regression) within each score phrase. RegC_WP and FixD100_WP represent within-participant components, whereas RegC_BP and FixD100_BP represent between-participant components. FixD100 was expressed in units of 100 ms.

Table S1b. Specifications of the regression-size and spatial-assignment sensitivity analyses.

Model	Distribution and link	Outcome	Specification	Purpose
M4 > 1-note regressions	Poisson; log	Regressive-fixation count excluding one-note regressions	EHST * R-accuracy + EHSN + PerfD + Score + (1 ∣ Participant) + (1 ∣ ScorePhrase)	Tests whether the focal M4 effects remain when one-note regressions are excluded.
M4 ≤ 0.6°	Poisson; log	Regressive-fixation count using a maximum fixation-to-note distance of 0.6°	EHST * R-accuracy + EHSN + PerfD + Score + (1 ∣ Participant) + (1 ∣ ScorePhrase)	Tests whether the focal M4 effects remain when fixation-to-note assignments are restricted to a maximum distance of 0.6°.
M4 ≤ 1.0°	Poisson; log	Regressive-fixation count using a maximum fixation-to-note distance of 1.0°	EHST * R-accuracy + EHSN + PerfD + Score + (1 ∣ Participant) + (1 ∣ ScorePhrase)	Tests whether the focal M4 effects remain when fixation-to-note assignments are restricted to a maximum distance of 1.0°.

Note. All models used a Poisson distribution with a log link. EHST = time-based eye–hand span; EHSN = note-based eye–hand span; PerfD = phrase performance duration; R-accuracy = rhythmic accuracy. EHST and EHSN were recalculated separately after applying each spatial-assignment threshold (≤ 0.6° and ≤ 1.0°). Score was effect-coded using sum-to-zero contrasts across BRIC, DEV, and KOE. All models included random intercepts for participant and score–phrase. N = 409 participant–phrase observations for all models.

https://doi.org/10.3390/jemr19050102

Table S2a. Means, standard deviations, and 95% confidence intervals for performance, oculomotor, anticipatory, and respiratory measures across the three musical scores.

**Measures**	***Mean***			***SD***			**95% CI**					
							LL			***UL***		
	**KOE**	**DEV**	**BRIC**	**KOE**	**DEV**	**BRIC**	**KOE**	**DEV**	**BRIC**	**KOE**	**DEV**	**BRIC**
RegC	3.15	4.91	2.64	2.25	3.30	2.11	2.71	4.28	2.33	3.59	5.54	2.95
RegD	260.1	335.78	303.73	96.00	142.96	143.00	240.22	307.41	280.81	279.99	364.14	326.64
RegSize	3.40	3.64	3.79	2.97	3.00	3.40	2.78	3.04	3.24	4.01	4.23	4.34
RegProp	0.08	0.10	0.05	0.05	0.05	0.07	0.07	0.09	0.07	0.09	0.11	0.08
EHST	0.98	1.27	1.09	0.18	0.39	0.35	0.94	1.20	1.04	1.01	1.35	1.14
EHSN	2.05	1.77	2.2	0.36	0.29	0.53	1.98	1.72	2.12	2.12	1.83	2.27
PerfD	18.02	26.00	16.66	3.14	7.38	5.94	17.4	24.61	15.79	18.63	27.9	17.53
R-accuracy	1.01	1.46	0.73	0.13	0.43	0.11	0.99	1.38	0.72	1.04	1.54	0.75
FixC	36.84	42.28	29.86	9.63	13.35	11.48	34.96	39.69	28.17	38.71	44.86	31.54
FixD	437.22	497.79	473.26	102.31	137.18	134.88	417.51	471.50	453.64	456.92	524.09	492.88
BlinkC	6.64	11.09	5.19	4.34	7.62	3.67	5.79	9.61	4.64	7.49	12.56	5.73
BlinkD	0.16	0.18	0.18	0.07	0.06	0.08	0.14	0.17	0.17	0.17	0.19	0.20
PupilD	3.42	3.44	3.46	0.42	0.4	0.41	3.34	3.36	3.4	3.5	3.52	3.52
BreathR	9.86	9.10	9.63	2.73	2.08	2.72	9.32	8.70	9.23	10.39	9.50	10.03

Note. CI = confidence interval; LL = lower confidence limit; UL = upper confidence limit; KOE = Koechlin's excerpt; DEV = *Makedonsko devojče* (Hristovski) score; BRIC = Briccialdi's excerpt; RegC = regressive-fixation count; RegD = regressive-fixation duration (ms); RegSize = mean regression size in notes ; RegProp = regression probability; EHST = time-based eye–hand span (s); EHSN = note-based eye–hand span; PerfD = phrase performance duration (s); R-accuracy = rhythmic accuracy; FixC = fixation count; FixD = mean fixation duration (ms); BlinkC = blink count; BlinkD = mean blink duration (s); PupilD = left-eye pupil dilation (mm); BreathR = number of respiratory cycles per minute.

https://doi.org/10.3390/jemr19050102

Table S2b. Medians and interquartile ranges for performance, oculomotor, anticipatory, and respiratory measures across the three musical scores.

	KOE	DEV	BRIC
RegC	3.00 [1.00-5.00]	4.00 [2.00-7.00]	2.00 [1.00-4.00]
RegD	254.00 [191.20-316.44]	295.30 [225.63-416.00]	268.67 [195.00-388.00]
RegSize	2.00 [1.22-4.81]	2.57 [1.50-4.44]	2.14 [1.00-6.00]
RegProp	0.07 [0.04-0.11	0.10 [0.06-0.14]	0.07 [0.04-0.11]
EHST	0.98 [0.85-1.07]	1.22 [0.98-1.53]	1.05 [0.82-1.31]
EHSN	2.03 [1.77-2.33]	1.76 [1.55-1.95]	2.15 [1.77-2.56]
PerfD	17.54 [15.73-20.46]	25.39 [20.78-31.09]	17.22 [14.34-20.17]
R-accuracy	1.00 [0.91-1.11]	1.39 [1.18-1.75]	0.77 [0.71-0.80]
FixC	36.50 [29.00-43.00]	39.00 [32.75-52.00]	29.00 [22.00-37.00]
FixD	441.72 [362.40-519.26]	508.86 [389.90-580.58]	470.01 [356.88-572.57]
BlinkC	6.00 [3.25-9.59]	8.84 [5.00-16.00]	5.00 [2.00-7.64]
BlinkD	0.15 [0.12-0.21]	0.18 [0.14-0.23]	0.19 [0.14-0.24]
PupilD	3.35 [3.15-3.76]	3.39 [3.16-3.73]	3.42 [3.20-3.77]
BreathR	10.33 [7.88-11.65]	8.83 [7.57-11.08]	9.17 [7.62-11.57]

Note. Values are presented as median [Q1–Q3], where Q1 and Q3 denote the first and third quartiles, respectively. KOE = Koechlin excerpt; DEV = *Makedonsko devojče* score; BRIC = Briccialdi excerpt; RegC = regressive-fixation count; RegD = regressive-fixation duration (ms); RegSize = regression size in notes ; RegProp = regression probability; EHST = time-based eye–hand span; EHSN = note-based eye–hand span; PerfD = phrase performance duration; R-accuracy = rhythmic accuracy; FixC = fixation count; FixD = mean fixation duration (ms); BlinkC = blink count; BlinkD = mean blink duration (s); PupilD = left-eye pupil dilation (mm); BreathR = number of respiratory cycles per minute.

https://doi.org/10.3390/jemr19050102

Table S3. Pearson correlations among participant-level mean oculomotor, respiratory, and performance measures during sight-reading.

	**RegC**	**RegD**	**RegSize**	**EHST**	**EHSN**	**PerfD**	**R-accuracy**	**FixC**	**FixD**	**BlinkC**	**BreathR**
RegC	1	-.566**	-.208	.680***	.260	.523**	.232	.931***	-.660***	.481**	-.377*
RegD	-.566**	1	-.065	-.323	-.397*	-.027	.002	-.576**	.723***	-.419*	.077
RegSize	-.208	-.065	1	-.092	.236	-.368	-.326	-.202	-.184	.257	.338
EHST	.680***	-.323	-.092	1	.583**	.702***	.556**	.697***	-.254	.450*	-.420*
EHSN	.260	-.397*	.236	.583**	1	-.094	-.036	.197	-.439*	.260	-.042
PerfD	.523**	-.027	-.368	.702***	-.094	1	.839***	.649***	.107	.382*	-.432*
R-accuracy	.232	.002	-.326	.556**	-.036	.839***	1	.390*	.240	.247	-.219
FixC	.931***	-.576**	-.202	.697***	.197	.649***	.390*	1	-.653***	.660***	-.405*
FixD	-.660**	.723***	-.184	-.254	-.439*	.107	.240	-.653***	1	-.604***	.065
BlinkC	.481**	-.419*	.257	.450*	.260	.382*	.247	.660***	-.604***	1	-.251
BreathR	-.377*	.077	.338	-.420*	-.042	-.432*	-.219	-.405*	.065	-.251	1

Note. RegC = regressive-fixation count; RegD = regressive-fixation duration (ms); RegSize = mean regression size in notes; EHST = eye–hand span in time; EHSN = eye–hand span in notes; PerfD = phrase performance duration; R-accuracy = rhythmic accuracy; FixC = fixation count; FixD = fixation duration; BlinkC = blink count; BreathR = respiratory cycles per minute. All correlations were computed from participant-level means, N = 28.
$^{*}p < .05$; $^{**}p < .01$; $^{***}p < .001$

Table S4. Variance inflation factors for the fixed effects in the maximum additive model

Predictor	Continuous predictor	VIF	Tolerance	Auxiliary R^2
PerfD	Yes	3.460	0.289	0.711
EHST	Yes	2.631	0.380	0.620
EHSN	Yes	2.503	0.399	0.601
R-accuracy	Yes	1.727	0.579	0.421
FixD	Yes	1.205	0.830	0.170
Score: DEV	No	1.829	0.547	0.453
Score: BRIC	No	1.641	0.609	0.391

Note. VIF = variance inflation factor. Tolerance = 1 − auxiliary R^2. PerfD = phrase performance duration; EHST = time-based eye-hand span; EHSN = eye–hand span in notes; R-accuracy = rhythmic accuracy; FixD = fixation duration. VIF values were calculated from the fixed-effect design matrix of the maximum additive model including performance duration, EHST, EHSN, R-accuracy, mean fixation duration, and score. Score was entered using effect coding; therefore, only two score contrasts are shown, with KOE represented implicitly by the sum-to-zero constraint. Continuous predictors were grand-mean centered.

https://doi.org/10.3390/jemr19050102

Table S5. Poisson rate models with log-transformed performance duration as an offset.

	M4Rate				SensFixDRate			
**Fixed effects**	**β**	**SE**	***t***	***p***	**β**	**SE**	***t***	***p***
Intercept	−1.733	0.107	−16.234	< .001	−1.764	0.076	−23.171	< .001
EHST	0.520	0.114	4.569	< .001	0.482	0.107	4.509	< .001
EHSN	0.140	0.085	1.643	.101	−0.005	0.084	−0.055	.956
R-accuracy	−0.936	0.328	−2.856	.005	−0.870	0.312	−2.785	.006
FixD (per 100 ms)					−0.305	0.030	−10.071	< .001
EHST × R-accuracy	0.898	0.392	2.289	.023	1.056	0.383	2.753	.006
Score: BRIC	−0.167	0.097	−1.719	.086	−0.129	0.078	−1.646	.101
Score: DEV	−0.053	0.111	−0.482	.630	0.017	0.089	0.187	.852
**Random effects**	**M4Rate**				**SensFixDRate**			
	**Variance**		**SD**		**Variance**		**SD**	
Participant	0.169		0.410		0.065		0.256	
Score-phrase	0.062		0.250		0.035		0.187	
Omnibus Score	***F***	**df**	***p***		***F***	**df**	***p***	
	2.293	2, 402	.102		1.465	2, 401	.232	

Note. β = unstandardized coefficient on the log-rate scale; SE = standard error; SD = standard deviation; *t* = test statistic for individual fixed-effect coefficients; *F* = omnibus test statistic; df = degrees of freedom; *p* = p-value; EHST = time-based eye–hand span; EHSN = note-based eye–hand span; PerfD = phrase performance duration FixD (per 100 ms) = mean fixation duration, scaled per 100 ms; R-accuracy = rhythmic accuracy. Models used a Poisson distribution with a log link and retained regressive-fixation count as the outcome, with log-transformed PerfD included as an offset. Continuous predictors were grand-mean centered. Score was effect-coded using sum-to-zero contrasts across BRIC, DEV, and KOE; KOE was the implicit level and not a reference category. Random intercepts were included for participant and score–phrase. *N* = 409 participant–phrase observations.

https://doi.org/10.3390/jemr19050102

Table S6. Binomial mixed-effects models of regression probability.

	SensRegProb				SensRegProbFixD			
**Fixed effects**	**β**	**SE**	***t***	***p***	**β**	**SE**	***t***	***p***
Intercept	-2.339	0.088	-26.655	< .001	-2.363	0.080	-29.592	< .001
EHST	0.496	0.133	3.730	< .001	0.495	0.131	3.784	< .001
EHSN	0.132	0.107	1.232	.219	0.047	0.107	0.437	.662
PerfD	0.016	0.010	1.629	.104	0.015	0.009	1.608	.109
R-accuracy	-0.805	0.393	-2.045	.042	-0.744	0.385	-1.934	.054
FixD (per 100 ms)					-0.143	0.032	-4.522	< .001
EHST× R-accuracy	1.052	0.419	2.512	.012	1.157	0.416	2.784	.006
Score: BRIC	-0.127	0.091	-1.397	.163	-0.108	0.085	-1.269	.205
Score: DEV	-0.013	0.099	-0.135	.892	0.019	0.093	0.206	.837
**Random effects**	**SensRegProb**				**SensRegProbFixD**			
	**Variance**		**SD**		**Variance**		**SD**	
Participant	0.095		0.308		0.071		0.266	
Score-phrase	0.045		0.211		0.037		0.193	
	**SensRegProb**				**SensRegProbFixD**			
Omnibus Score F	***F***	***df***	***p***		***F***	***df***	***p***	
	1.261	2, 401	.284		0.853	2, 400	.427	

Note. β = unstandardized coefficient on the logit scale; SE = standard error; SD = standard deviation; *t* = test statistic for individual fixed-effect coefficients; df = degrees of freedom; *p* = p-value; EHST = time-based eye–hand span; EHSN = note-based eye–hand span; FixD (per 100 ms) = mean fixation duration, scaled per 100 ms; PerfD = phrase performance duration; R-accuracy = rhythmic accuracy. Models used a binomial distribution with a logit link and treated regressions as successes out of the total number of valid consecutive fixation comparisons, which was specified as the binomial size. Continuous predictors were grand-mean centered. Score was effect-coded using sum-to-zero contrasts across BRIC, DEV, and KOE; KOE was the implicit level and not a reference category. Random intercepts were included for participant and score-phrase. *N* = 409 participant–phrase observations.

https://doi.org/10.3390/jemr19050102

Table S7. Linear mixed-effects model for log-transformed relative performance duration: exploratory temporal-compensation analysis.

**Fixed effects**					
**Predictor**	***b***	**SE**	***t***	**df**	***p***
Intercept	0.151	0.052	2.939	14.264	.011
Score: BRIC	-0.055	0.063	-0.876	11.767	.398
Score: DEV	0.092	0.072	1.269	11.881	.229
RegC_WP	0.022	0.003	6.872	371.700	< .001
FixD100_WP	0.033	0.007	4.472	363.330	< .001
RegC_BP	0.054	0.010	5.169	28.317	< .001
FixD100_BP	0.085	0.021	4.025	25.415	< .001
EHST	0.213	0.024	8.977	388.320	< .001
EHSN	-0.256	0.018	-14.323	387.890	< .001
RegC_WP × FixD100_WP	-0.002	0.003	-0.836	368.970	.404
**Random effects**	**Variance**		**SD**		
Participant	0.007		0.085		
Score-phrase	0.033		0.182		
Residual	0.012		0.111		
**Likelihood-ratio test**	**LR χ^2**		**df**		***p***
Interaction model vs. additive model	0.673		1		.412

Note. b = unstandardized coefficient; SE = standard error; SD = standard deviation; t = test statistic for individual fixed-effect coefficients; df = approximate Satterthwaite degrees of freedom; p = p-value; LR χ^2 = likelihood-ratio chi-square statistic. The outcome was the natural logarithm of the ratio between observed phrase duration and MIDI-reference phrase duration. Fixed-effect estimates and random-effect parameters are reported from the model fitted using restricted maximum likelihood. The likelihood-ratio test compared the interaction model with the corresponding additive model, both fitted using maximum likelihood. EHST = time-based eye–hand span; EHSN = note-based eye–hand span; RegC = regressive-fixation count; FixD100 = mean fixation duration expressed in units of 100 ms. RegC_WP and FixD100_WP represent within-participant components centered around each participant's mean, whereas RegC_BP and FixD100_BP represent between-participant components. EHST and EHSN were grand-mean centered. Score was effect-coded using sum-to-zero contrasts across BRIC, DEV, and KOE; KOE was the implicit level and not a reference category. Random intercepts were included for participant and score–phrase. The analysis included 409 participant–phrase observations from 28 participants and 15 score–phrase units.

Table S8. Poisson mixed-effects sensitivity models for spatial-assignment thresholds and regression-size exclusion.

	**M4 ≤ 0.6°**	**M4 ≤ 1.0°**	**M4 > 1-note regressions**

**Fixed Effects**	**β**	**SE**	***t***	***p***	**β**	**SE**	***t***	***p***	**β**	**SE**	***t***	***p***
Intercept	0.804	0.107	7.515	< .001	1.041	0.102	10.212	< .001	0.329	0.141	2.337	.020
EHST	0.712	0.155	4.593	< .001	0.623	0.137	4.549	< .001	0.602	0.196	3.071	.002
EHSN	0.077	0.123	0.626	.532	0.131	0.112	1.166	.244	0.147	0.157	0.937	.349
Score BRIC	-0.164	0.088	-1.866	.063	−0.182	0.087	−2.103	.036	-0.193	0.163	-1.184	.237
Score DEV	0.038	0.096	0.396	.693	−0.026	0.095	−0.271	.786	0.055	0.178	0.307	.759
PerfD	0.053	0.010	4.852	< .001	0.058	0.010	5.651	< .001	0.047	0.015	3.054	.002
R-accuracy	−0.295	0.434	-0.679	.498	−0.330	0.399	−0.827	.408	0.603	0.607	0.995	.321
EHST × R-accuracy	1.249	0.549	2.275	.023	1.171	0.463	2.529	.012	1.198	0.615	1.947	.052
**Random Effects**	**M4 ≤ 0.6°**				**M4 ≤ 1.0°**				**M4 > 1-note regressions**			
	**Variance**		**SD**		**Variance**		**SD**		**Variance**		**SD**	
Participant	0.207		0.455		0.181		0.425		0.156		0.396	
Score-phrase	0.035		0.188		0.038		0.196		0.166		0.407	
Omnibus Score	***F***	**df**	***p***		***F***	**df**	***p***		***F***	**df**	***p***	
	1.796	2, 401	.167		2.930	2, 401	.055		0.721	2, 401	.487	

Note. β = unstandardized coefficient on the log scale; SE = standard error; SD = standard deviation; *t* = test statistic for individual fixed-effect coefficients; *F* = omnibus test statistic for Score; df = degrees of freedom; *p* = p-value. EHST = time-based eye–hand span; EHSN = note-based eye–hand span; PerfD = phrase performance duration; R-accuracy = rhythmic accuracy. All models used a Poisson distribution with a log link for regressive-fixation count. Continuous predictors were grand-mean centered before model fitting. Score was effect-coded using sum-to-zero contrasts across BRIC, DEV, and KOE; KOE was therefore the implicit level and not a reference category. Random intercepts were included for participant and score–phrase. M4 ≤ 0.6° and M4 ≤ 1.0° used regressive-fixation counts based on maximum fixation-to-note distances of 0.6° and 1.0°, respectively. EHST and EHSN were recalculated separately after applying each spatial-assignment threshold. The M4 > 1-note regressions model repeated M4 after excluding one-note regressions. N = 409 participant–phrase observations for all models.

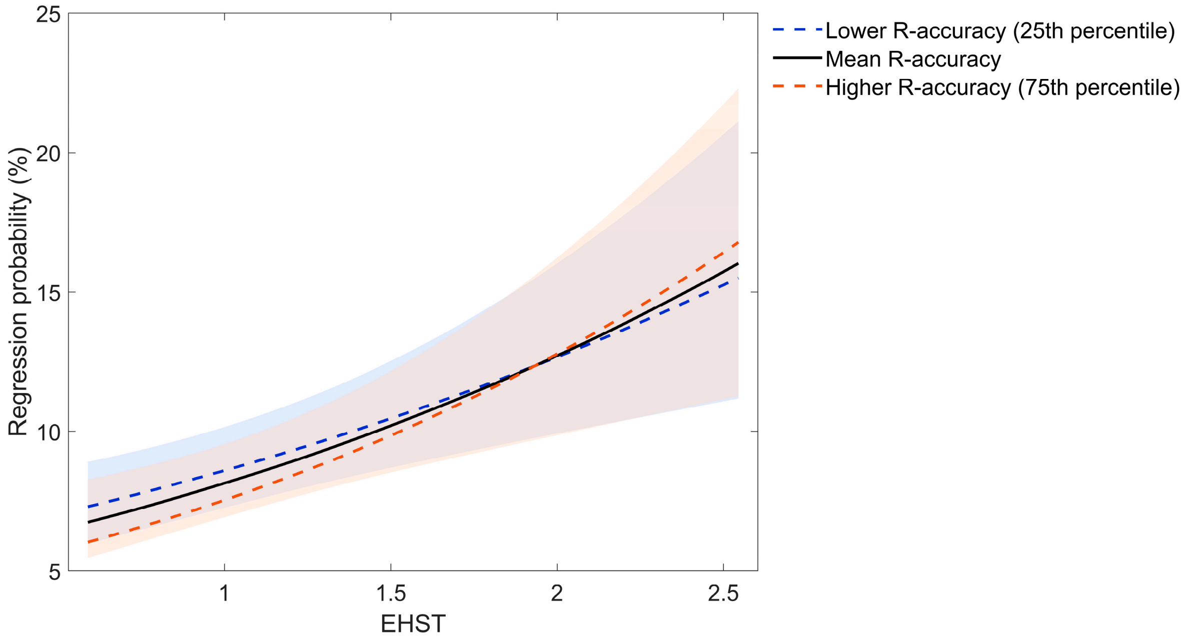

Figure S1. Predicted regression probability as a function of EHST and R-accuracy. Lines represent model-based predictions at the 25th percentile, mean, and 75th percentile of R-accuracy. Shaded areas indicate 95% confidence intervals for the 25th- and 75th-percentile curves. Predictions are derived from the supplementary binomial mixed-effects model (SensRegProb). EHST = time-based eye–hand span; R-accuracy = rhythmic accuracy.

https://doi.org/10.3390/jemr19050102

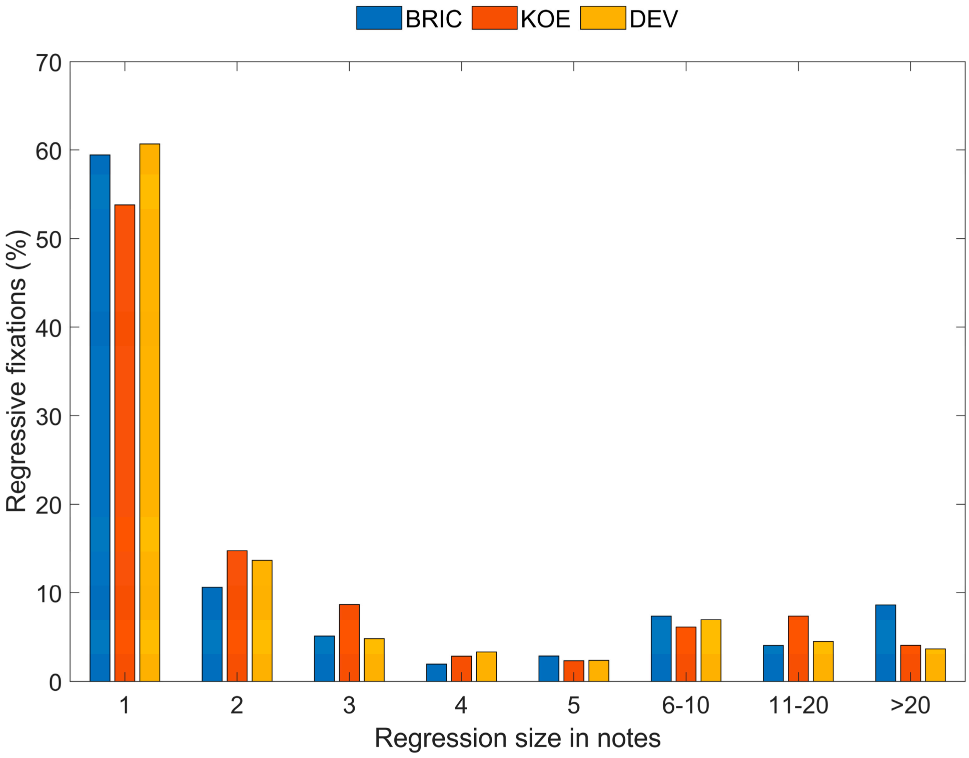

Figure S2. Distribution of regression sizes by score. Bars show the percentage of regressive-fixation events within each score falling into each regression-size category. Regression size was defined as the distance, in notes, between the score location assigned to the fixation immediately preceding the regression and the score location assigned to the regressive fixation. Regression sizes from one to five notes are shown individually, whereas larger regressions are grouped into 6–10, 11–20, and >20-note categories.
